# Diesel exhaust impairs TREM2 to dysregulate neuroinflammation

**DOI:** 10.1186/s12974-020-02017-7

**Published:** 2020-11-22

**Authors:** Hendrik J. Greve, Christen L. Mumaw, Evan J. Messenger, Prasada R. S. Kodavanti, Joyce L. Royland, Urmila P. Kodavanti, Michelle L. Block

**Affiliations:** 1 Department of Pharmacology and Toxicology, The Stark Neurosciences Research Institute, Indiana University School of Medicine, 320 West 15th Street, NB 214D, Indianapolis, IN 46202 USA; 2 Neurological and Endocrine Toxicology Branch, Public Health and Integrated Toxicology Division, Center for Public Health and Environmental Assessment, Office of Research and Development, U.S. Environmental Protection Agency, Research Triangle Park, NC USA; 3 Integrated Systems Toxicology Division, National Health and Environmental Effects Research Laboratory, Office of Research and Development, U.S. Environmental Protection Agency, Research Triangle Park, NC USA; 4 Cardiopulmonary and Immunotoxicology Branch, Public Health and Integrated Toxicology Division, U.S. Environmental Protection Agency, Research Triangle Park, NC USA

**Keywords:** Air pollution, Brain, Microglia, Neuroinflammation, TREM2, Alzheimer’s disease, Disease-associated microglia

## Abstract

**Background:**

Air pollution has been linked to neurodegenerative diseases, including Alzheimer’s disease (AD), and the underlying neuroimmune mechanisms remain poorly understood. TREM2 is a myeloid cell membrane receptor that is a key regulator of disease-associated microglia (DAM) cells, where loss-of-function *TREM2* mutations are associated with an increased risk of AD. At present, the basic function of TREM2 in neuroinflammation is a point of controversy. Further, the impact of air pollution on TREM2 and the DAM phenotype is largely unknown. Using diesel exhaust (DE) as a model of urban air pollution exposure, we sought to address its impact on TREM2 expression, the DAM phenotype, the association of microglia with the neurovasculature, and the role of TREM2 in DE-induced neuroinflammation.

**Methods:**

WYK rats were exposed for 4 weeks to DE (0, 50, 150, 500 μg/m^3^) by inhalation. DE particles (DEP) were administered intratracheally once (600 μg/mouse) or 8 times (100 μg/mouse) across 28 days to male mice (*Trem2*^*+/+*^, *Trem2*^*−/−*^, PHOX^*+/+*^, and PHOX^*−*/*−*^).

**Results:**

Rats exposed to DE exhibited inverted-U patterns of *Trem2* mRNA expression in the hippocampus and frontal cortex, while TREM2 protein was globally diminished, indicating impaired TREM2 expression. Analysis of DAM markers *Cx3Cr1*, *Lyz2*, and *Lpl* in the frontal cortex and hippocampus showed inverted-U patterns of expression as well, supporting dysregulation of the DAM phenotype. Further, microglial-vessel association decreased with DE inhalation in a dose-dependent manner. Mechanistically, intratracheal administration of DEP increased *Tnf* (TNFα), *Ncf1* (p47^PHOX^), and *Ncf2* (p67^PHOX^) mRNA expression in only *Trem2*^*+/+*^ mice, where *Il1b* (IL-1β) expression was elevated in only *Trem2*^*−/−*^ mice, emphasizing an important role for TREM2 in DEP-induced neuroinflammation.

**Conclusions:**

Collectively, these findings reveal a novel role for TREM2 in how air pollution regulates neuroinflammation and provides much needed insight into the potential mechanisms linking urban air pollution to AD.

**Supplementary Information:**

The online version contains supplementary material available at 10.1186/s12974-020-02017-7.

## Introduction

Air pollution is rapidly increasing in prevalence in multiple countries and is a major contributor to the global burden of disease [1]. More specifically, components of urban air pollution are associated not only with pulmonary and cardiovascular diseases, but also with central nervous system (CNS) diseases and conditions, including Alzheimer’s disease (AD) [2–4], cognitive decline [5–8], and dementia [9, 10]. Accumulating research with murine models demonstrates that components of air pollution impact the CNS and AD-like pathology [11, 12]. However, at present, the mechanisms underlying the link between air pollution and AD remain poorly understood.

Microglia, the myeloid cell of the brain parenchyma, function as immune sentinels and are a prominent source of neuroinflammation in the CNS during disease [13, 14], which is implicated as a central mechanism of many neurodegenerative diseases, including AD [15]. Increasing reports indicate that multiple forms of air pollution, including diesel exhaust (DE), trigger neuroinflammation and augment microglial pro-inflammatory responses both in vitro and in vivo [12, 16]. These studies, and others, have led to the neuroinflammation hypothesis, which posits that the air pollution-induced elevation in cytokines and reactive oxygen species (ROS) in the CNS may mediate the detrimental effects of air pollution on the brain [17]. While neuroinflammation appears to be a common link between AD and air pollution, how exposure to air pollution triggers and regulates neuroinflammation is poorly understood.

Microglia have long been implicated in AD pathology [18], where their roles in the clearance of amyloid and the associated mechanisms have also become increasingly highlighted [19]. Recently, genome-wide association studies have identified several triggering receptor expressed on myeloid cells 2 (*TREM2*) mutations that are risk factors for AD [20, 21]. While first described as anti-inflammatory, more recent studies have demonstrated that TREM2 plays more complex roles during neuroinflammation [22–24]. Murine studies have revealed that TREM2 regulates microglia-mediated responses to neurodegenerative processes [22], such as the chemotactic microglial response to amyloid plaques [25], the pro-inflammatory response of microglia [23], microglial survival [26], and the disease-associated microglia (DAM) phenotype [27]. The DAM phenotype was identified as a microglia subset that performs the beneficial roles of restricting amyloid plaque development and removing protease-resistant pathogenic proteins [27]. Importantly, reports indicate that TREM2 is essential for the second stage of the DAM phenotype [23, 27, 28]. At present, the impact of air pollution on TREM2 and the DAM phenotype is unknown.

The neurovasculature plays key roles in the communication of the periphery to the brain parenchyma during both homeostasis and disease [29], including AD [30]. The neurovasculature has also been implicated in the communication between peripheral immune responses and the brain parenchyma [31]. In fact, peripheral inflammation has been shown to regulate microglial association with the neurovasculature [31, 32]. Importantly, components of air pollution have been shown to impact the neurovasculature, including the blood-brain barrier (BBB), resulting in damaged vasculature and a disrupted barrier [33, 34]. However, while there are clear neurovascular effects, the impact of air pollution on the cellular interactions between microglia and the neurovasculature is not well understood.

Currently, the mechanisms underlying how urban air pollution regulates neuroinflammation and why urban air pollution is associated with AD are largely unknown. To begin to address these issues, we employed murine models of DE exposure to understand the impact of urban air pollution on TREM2 expression, the DAM phenotype, and the microglial association with the neurovasculature during neuroinflammation. Further, we used *Trem2*^*+/+*^ and *Trem2*^*−/−*^ mice to explore the mechanistic role of TREM2 in DE particle (DEP)-induced neuroinflammation.

## Materials and methods

### Reagents

Standard reference material (SRM) 2975 diesel particulate matter (industrial forklift) was obtained from the National Institute for Standards and Technology (Gaithersburg, MD, USA). 2-(4-Amidinophenyl)-6-indolecarbamidine dihydrochloride (DAPI) was purchased from Roche Diagnostics (Indianapolis, IN, USA). The rabbit anti-ionized calcium-binding adaptor molecule 1 (IBA-1) antibody was purchased from Wako (Richmond, VA, USA), the rat anti-mouse CD31 antibody from BD Biosciences (Franklin Lakes, NJ, USA), and the mouse anti-rat CD31 from Invitrogen (Carlsbad, CA, USA). LPS (strain O111:B4) was purchased from EMD Chemicals (Gibbstown, NJ, USA). All other reagents were purchased from Sigma-Aldrich (St. Louis, MO, USA).

### Animals

Both mice and rats were used in this study, as they have been shown to exhibit similar toxicological and pulmonary responses to DE exposure [35, 36]. Twelve-to-fourteen-week-old male Wistar Kyoto (WKY) rats (*n* = 7 per group) were obtained from Charles River Laboratories Inc. (Raleigh, NC, USA). Seven-to-eight-week-old male *Cybb*^*+/+*^ (PHOX^+/+^), *Cybb*^*−/−*^ (PHOX^*−/−*^), *Trem2*^*+/+*^, and *Trem2*^*−/−*^ mice (*n* = 3–6 per group) were purchased from Jackson Laboratory (Bar Harbor, ME, USA), where the genetically modified mouse strains were both on a C57Bl/6J background. The PHOX^−/−^ mice (Jax – 002365) lack the functional catalytic subunit of the nicotinamide adenine dinucleotide phosphate (NADPH) oxidase complex, gp91^PHOX^. NADPH oxidase is an inducible electron transport system in phagocytic cells that is responsible for the generation of the respiratory burst. PHOX^*−/−*^ mice are unable to generate extracellular superoxide in response to lipopolysaccharide or other immunological stimuli. *Trem2*^*−/−*^ mice (Jax – 027197) were generated with CRISPR/Cas9 knock-out of *Trem2*, where there is a NHEJ-generated 175-bp deletion that introduces a stop codon at amino acid 17. The TREM2 protein is a member of a receptor signaling complex that activates the macrophage immune response, where mutations are associated with increased risk to AD.

Animals were housed in an AAALAC-accredited housing facility maintained at 20–24 °C on a 12-h light/dark cycle. Animals were acclimated to the housing facility for 1 week prior to the beginning of studies. Animals were singly housed in microisolator cages with food and water provided ad libitum. All experiments were approved by the IACUC (IUSM 11327 and EPA 12-06-002) and were in accordance with the National Institutes of Health guidelines for housing, breeding, and experimental use. All animals were treated humanely and with regard for alleviation of suffering.

### Animal exposures and experimental design

#### Diesel exhaust inhalation exposure

In order to determine how air pollution exposure may impact TREM2, WKY rats were exposed to DE by whole-body inhalation. We have previously explored the neuroimmune impact of DE inhalation in WKY rats [37, 38], and there is a wealth of physiological and toxicological information on the effects of air pollution on WKY rats, making them particularly useful in examining new effects of air pollution on health and disease [39–42]. Petroleum diesel fuel with ultra-low sulfur (< 15 ppm) (Red Star Oil, Raleigh, NC, USA) was used to generate diesel exhaust (DE) by a single-cylinder, air-cooled, direct injection, 320-cm^3^ Yanmar L70 diesel engine (Adairville, GA, USA) ran at 5.8 hp (4.3 kW) continuous load, 3600 rpm, and Pramac E3750 generator (Marietta, GA, USA) [43]. WKY rats were exposed to whole DE in whole-body chambers for 4 h/day, 5 days/week, for 1 month to filtered air (FA, 0 μg/m^3^) control or DE (50 μg/m^3^, 150 μg/m^3^, or 500 μg/m^3^). These concentrations represent an occupational exposure and are an established model of air pollution-induced cardiopulmonary system [36, 39, 44] and CNS damage [37].

#### Intratracheal (IT) diesel exhaust particle administration

To examine the functional significance of TREM2 loss in DEP-induced neuroinflammation, an intratracheal (IT) model of DEP administration was used in mice, as we have previously reported that DEP IT administration has a CNS TNFα response similar to DE inhalation [37]. The use of an intratracheal administration model allows for the specific examination of how the particulate component of diesel exhaust in the lung may impact neuroinflammation [37]. For the acute model, mice received either one dose of DEP (SRM 2975; 12 mg/mL, 50 μL; 600 μg/mouse) suspended in vehicle (phosphate-buffered saline + 0.05% Tween 80, pH 7.4) or vehicle alone as a control, as previously described [37, 45]. Tissues were collected 24 h after administration. For the month-long model, mice received either DEP (2 mg/mL, 50 μL; 100 μg/mouse) or vehicle by IT route 2 times per week for 4 weeks, as previously reported [16], and tissues were collected 24 h after final administration.

#### Intraperitoneal LPS injection

To explore how TREM2 might affect another type of peripheral immune response that triggers neuroinflammation, LPS was used. Mice received a single intraperitoneal (IP) injection of LPS (5 mg/kg) or vehicle (0.9% saline), as previously reported [46, 47]. Mice were euthanized 3 h after LPS treatment, and brain tissue was collected.

#### Brain tissue collection

In each model, mice or rats were euthanized and the right hemisphere of each brain was fixed in a 4% paraformaldehyde solution and cryopreserved in 30% sucrose, while the left hemisphere was dissected into relevant brain regions (hippocampus and cortex) and snap frozen, where all tissues were stored at − 80 °C until analysis.

#### Adult microglia isolation

Microglia from adult mouse brains were isolated as previously described [48–50]. Briefly, mice were anesthetized 24 h post-treatment and were perfused with cold PBS. Microglia were isolated utilizing the Miltenyi Neural Tissue Dissociation Kit (P) (Miltenyi Biotec, San Diego, CA) according to the manufacturer’s instructions.

#### Lung sample collection and processing

Bronchoalveolar lavage fluid (BALF) was collected from mice by lavaging the lung two times with 1 mL of sterile Hank’s balanced salt solution (HBSS) without Ca^2+^ or Mg^2+^. BALF was centrifuged at 1500×*g* for 10 min at 4 °C, and the supernatant from the first lavage was frozen at − 80 °C for biochemical analysis. Both lavages were combined, and the resulting cell pellets were resuspended in 250 μL of sterile PBS. The total protein in the supernatant was determined by bicinchoninic acid assay (Thermo Fisher Scientific, Rockford, IL, USA). Total cells were determined by using a TC-10 automated cell counter (Bio-Rad, Hercules, CA, USA). Cells were centrifuged onto slides and differentially stained and quantified as previously described [39].

Lungs were prepared for histopathology as previously described [51]. Briefly, after BALF collection, lungs were inflated through the trachea with paraformaldehyde (PFA)/optimal cutting temperature compound (OCT) mixture, tied off, and drop fixed in 4% PFA overnight at 4 °C. Lungs were transferred to 30% sucrose at 4 °C for 24 h before embedding in OCT and sectioning on a cryostat (Leica Biosystems, Buffalo Grove, IL). Five-micrometer sections were placed directly on slides and allowed to dry before undergoing H&E staining. Images of lungs were acquired on a Leica DM 2500 microscope (Leica Biosystems).

#### Quantitative reverse transcription-polymerase chain reaction (RT-qPCR)

Total RNA was extracted from mouse left hemisphere and rat cortex and hippocampal tissues with TRIzol (Thermo Scientific-Invitrogen), according to the manufacturer’s instructions. The extracted RNA was then treated with Ambion DNaseI (Thermo Scientific-Invitrogen). Reverse transcription of RNA (1.0 μg/sample) was performed with Maxima First Strand cDNA Synthesis Kit (Thermo Fisher Scientific). PowerUp Sybr Green Master Mix (Life Technologies) and 500 nM forward and reverse primers were used in quantitative PCR on a Viia7 (Life Technologies) RT-PCR system. Cycling parameters were 1 cycle of 95 for 15 s, 95 for 5 s, and 56 for 20 s for 40 cycles, and a melting curve measurement of 5 s 0.5 incremental temperature increases from 60 to 95. Primer sequences are listed in Tables S1 and S2. For examination of *Nfel2l* and *Hmox1* in rat brains, TaqMan probes were used according to the manufacturer’s instructions (Applied Biosystems, Foster City, CA). Relative quantities of mRNA transcripts were calculated using the 2^−ΔΔCT^ method.

#### Immunohistochemistry

The right hemisphere of each mouse or rat brain was fixed in a 4% PFA solution and cryopreserved in 30% sucrose. Forty-micrometer coronal sections were taken through the hippocampus using a freezing stage microtome (Microm HM 450, Thermo Scientific, Waltham, MA, USA). For immunofluorescent assessment of microglia-neurovascular association, free-floating sections were first washed for 10 min in 0.1% Triton X-100 in phosphate-buffered saline (PBST) at room temperature. Antigen retrieval was performed by incubating sections in 10 mM sodium citrate (pH = 6.0) with 0.5% Tween 20 at 85 °C for 15 min and then placing at room temperature for 30 min, as previously described [23]. Sections were then blocked in 5% normal goat serum (NGS) in PBST for 1 h at room temperature. Samples were transferred into primary antibody solution, consisting of primary antibodies and blocking solution, at 4 °C overnight. The primary antibodies used were polyclonal rabbit anti-ionized binding adaptor molecule-1 (Iba-1, 1:1000) antibody polyclonal mouse anti-CD31 (1:500) antibody, and rat anti-mouse CD31 (1:500). Slices were washed 3 times in PBST for 10 min, then incubated in a blocking solution containing secondary antibodies for 1 h, protected from light. The secondary antibodies used were Alexa Fluor 568 goat anti-mouse IgG, Alexa Fluor 647 goat anti-rat IgG, and Alexa Fluor 488 goat anti-rabbit IgG (Thermo Fisher Scientific). Slices were washed 3 times in PBST for 10 min, then incubated in DAPI for 5 min. Slices were washed 3 times in PBS. Images were acquired using a Nikon A1R confocal microscope (Nikon Instruments, Melville, NY). Microglia-neurovascular association was measured, as previously described [32]. Z-stacks (1 μm steps) were acquired through the CA1 hippocampus in 4 slices per animal from Bregma − 3.14 to − 3.50 mm for rats and − 2.56 to − 2.92 mm for mice. Images were blinded, and the number of vessel-associated microglia and non-vessel-associated microglia was counted using ImageJ software (NIH).

#### Enzyme-linked immunosorbent assay (ELISA)

Protein was extracted from the left hemisphere frontal cortex of the WKY rats by homogenization with a motorized pestle in Cytobuster Protein Extraction Reagent (EMD Chemicals, Darmstadt, Germany) with 10 μL/mL HALT protease inhibitor, phosphatase inhibitor, and 10 μL/mL EDTA, as previously described [38]. The protein concentration was determined using a BCA protein assay (Thermo Fisher Scientific). TREM2 was measured in brain homogenate using commercially available ELISA kits (MyBioSource, San Diego, CA, USA).

#### Microglia morphology quantification

To assess changes in cell volume, images of IBA-1 and fluorescent staining in the hippocampus were acquired as Z-stacks (1 μm steps) using a Nikon A1R confocal microscope and a × 40 objective (Nikon Instruments, Melville, NY). Three hippocampus slices were analyzed per mouse. Hypertrophic microglia were defined as cells with a volume greater than 600 μM^3^ and counted using the NIS Elements program (Nikon Instruments, Melville, NY). Microglia morphology and branch analysis was performed as previously described using Nikon NIS Elements analysis software [52, 53].

### Statistical analysis

Data are expressed as the mean ± SEM and were analyzed by one- or two-way analysis of variance (ANOVA) using GraphPad Prism (GraphPad Prism, San Diego, CA, USA). Mean differences were analyzed by Bonferroni’s post hoc analysis or Student *t* test, where appropriate. A *p* value of *p* < 0.05 was considered statistically significant.

## Results

### Diesel exhaust exposure induces neuroinflammation and impairs TREM2 expression

**Fig. 1:**
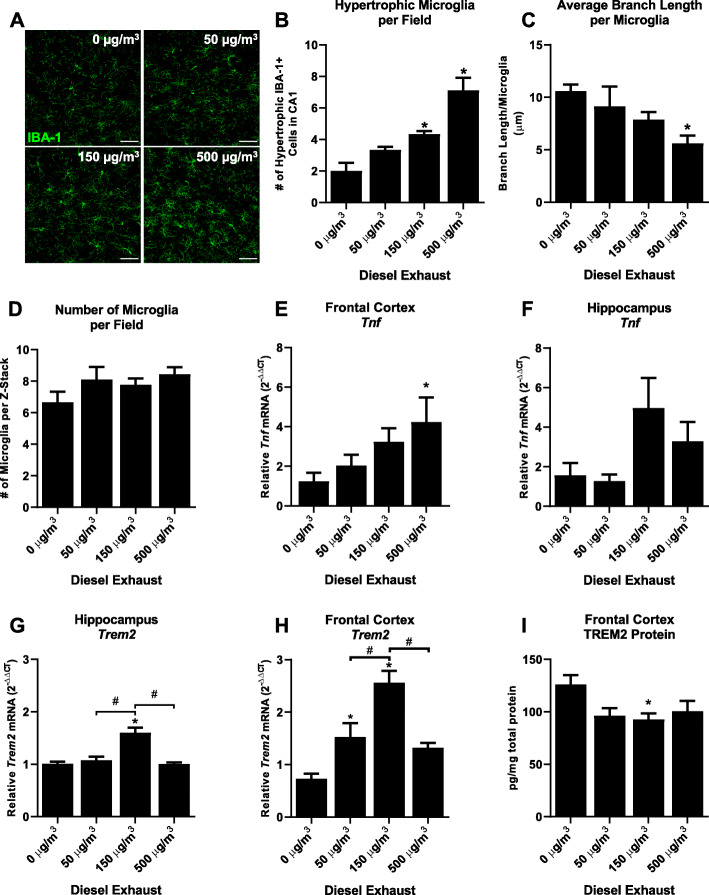
Diesel exhaust impairs TREM2 expression during neuroinflammation. WKY rats were exposed to diesel exhaust (0, 50, 150, or 500 μg/m^3^) by inhalation for 4 weeks. Representative images taken at × 40 of IBA-1 immunofluorescence in the hippocampus are shown (*n* = 3). The scale bar depicts 50 μm (**a**). In the hippocampus, the number of microglia with a volume greater than 600 μm (hypertrophic microglia) (**b**), the average branch length per microglia (**c**), and the total number of microglia (**d**) were counted in the slices were assessed. *Tnf* (TNFα) mRNA levels were measured in the frontal cortex (**e**) and hippocampus (**f**). *Trem2* mRNA levels were assessed in the hippocampus (**g**) and frontal cortex (**h**). mRNA levels were analyzed by qRT-PCR and normalized to *Gapdh* using the 2^−ΔΔCT^ method. **i** Frontal cortex TREM2 protein levels were analyzed by ELISA. Data are reported as the mean ± SEM. *p < 0.05 when compared to control. ^#^*p* < 0.05 compared between exposure groups (*n* = 7)

To begin to understand more about how urban air pollution could affect CNS diseases like AD, we first assessed whether DE-induced neuroinflammation was associated with TREM2 abnormalities, as inactivating mutations are a risk factor for AD [20, 21, 54]. Consistent with prior reports documenting DE-induced neuroinflammation without changes in microglial cell numbers [37], we observed changes in microglial morphology in the CA1 hippocampus of WYK rats exposed to DE, as measured by microglial branch analysis and hypertrophic microglia numbers (Fig. 1 a–c, *p* < 0.05), with no significant changes in total microglia cell numbers (Fig. 1 d, *p* > 0.05). mRNA expression of the pro-inflammatory cytokine *Tnf* (TNFα) increased at the highest level of exposure in the frontal cortex but not in the hippocampus (Fig. 1 b, c, *p* < 0.05), further demonstrating that month-long DE inhalation triggered neuroinflammation. We also examined the expression of TREM2, which has important roles in microglial physiology, where *TREM2* mutations have been implicated in AD [20, 21]. Exposure to month-long DE inhalation induced an inverted-U pattern of *Trem2* mRNA expression in both the hippocampus and the frontal cortex (Fig. 1 d, e, *p* < 0.05), supporting that air pollution exposure can disrupt *Trem2* gene expression. Analysis of TREM2 protein expression in the frontal cortex revealed a reduction in TREM2 levels, which was significant at 150 μg/m^3^ DE (Fig. 1 f, *p* < 0.05). We next sought to examine whether month-long DE exposure impaired expression of key genes in the antioxidant system, *Hmox1* and *Nfel2l*, where mRNA expression of these genes were unchanged in any exposure group (Supplemental Figure 1A & B, *p* > 0.05). Taken together, these findings indicate that during month-long DE-induced neuroinflammation, TREM2 expression is disrupted in the hippocampus and cortex, two brain regions affected in AD.

### Diesel exhaust exposure dysregulates the disease-associated microglia (DAM) phenotype

**Fig. 2:**
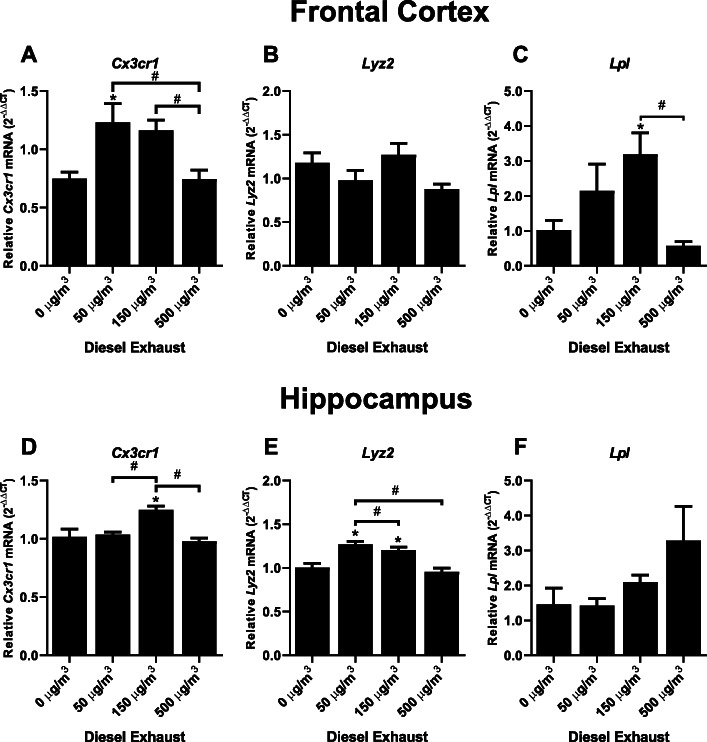
Diesel exhaust dysregulates markers of disease-associated microglia. WKY rats were exposed to diesel exhaust (0, 50, 150, or 500 μg/m^3^) by inhalation for 4 weeks. Changes in gene expression of the disease-associated microglia markers were analyzed by qRT-PCR. Stage 1 DAM markers *Cx3cr1* and *Lyz2* were analyzed in the frontal cortex (**a**, **b**) and the hippocampus (**d**, **e**). The stage 2 DAM marker *Lpl* was analyzed in the frontal cortex (**c**) and hippocampus (**f**). Data are reported as the mean ± SEM. *p < 0.05 when compared to control. ^#^*p* < 0.05 compared between exposure groups (*n* = 7)

In an effort to explore the consequences of impaired TREM2 expression in response to DE, we next investigated the effects of DE on the expression of other DAM markers. TREM2 regulates the stage 2 DAM cell phenotype, which is responsible for the beneficial plaque clearing phenotype in microglia [27, 28]. More specifically, the DAM phenotype is characterized in part by changes in gene expression markers: *Cx3cr1* decrease (stage 1), *Lyz2* (lysozyme) increase (stage 1), and *Lpl* increase (stage 2). In the cortex, both *Cx3cr1* and *Lpl* showed inverted-U patterns of expression (Fig. 2 a, c, *p* < 0.05), but the expression of *Lyz2* was unaffected (Fig. 2 b, *p* > 0.05), while *Cx3cr1* and Lyz2 showed inverted-U patterns of expression in the hippocampus (Fig. 2 d–f, *p* < 0.05) in response to 1-month DE exposure. These data suggest that exposure to DE may dysregulate the DAM 1 and 2 phenotypes in a concentration- and region-dependent manner, which have been hypothesized to play beneficial roles during AD, such as restricting plaque development and clearing pathogenic amyloid beta from the brain [27].

### Diesel exhaust exposure impairs microglia-neurovascular association

The physical association of microglia with the neurovasculature has been implicated in the CNS parenchymal consequences of peripheral immune responses, and air pollution has long been known to affect the BBB [31, 33], emphasizing the importance of the neurovasculature in these processes. To test the effect of month-long DE inhalation on the BBB, we examined mRNA expression of *Aqp4* (Aquaporin-4, expressed on the astrocytic endfeet at the BBB), *Mdr1b* (p-Glycoprotein, a key efflux transporter at the BBB) and *Vcam1* and *Icam1* (VCAM-1, ICAM-1, two cellular adhesion molecules that have important roles in immune cell trafficking). Analysis of mRNA expression of these markers demonstrated no change in their expression at any concentration of DE exposure (Supplemental Figure 2, *p* > 0.05).

**Fig. 3:**
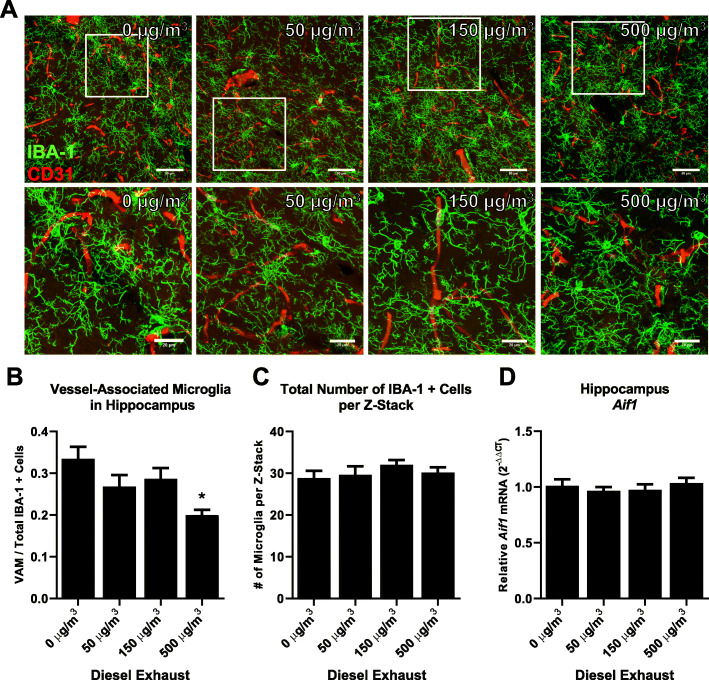
Diesel exhaust impairs microglial association with the neurovasculature. WKY rats were exposed to diesel exhaust (0, 50, 150, or 500 μg/m^3^) by inhalation for 4 weeks. The changes in the association of microglia cell bodies with the neurovasculature were assessed with confocal images of microglia (IBA-1, green) and vascular endothelial cells (CD31, red) in the hippocampus (4 slices per brain). Representative maximum intensity projection images are shown. The scale bar depicts 50 μm (top) or (20 μm) bottom (*p* < 0.05, *n* = 3) (**a**). The number of vessel-associated microglia in the CA-1 hippocampus was counted (*p* < 0.05, *n* = 3) (**b**). The total number of IBA-1+ cells in the confocal images was counted (*p* < 0.05, *n* = 3) (**c**). mRNA analysis of *Aif1* (IBA-1) expression demonstrates no change in expression of IBA-1 in the hippocampus of WKY rats (*p* < 0.05, *n* = 7). Data are reported as the mean ± SEM. *p < 0.05 when compared to control

We next explored whether DE might affect the direct interaction of microglia with the neurovasculature. Immunostaining of IBA-1 (microglia) and CD31 (endothelial cells) and Z-stack acquisition through the CA1 hippocampus revealed an altered association of the microglia with the vessels in the CA1 hippocampus region (Fig. 3a, representative images are shown). Quantification of the number of microglia showed a decrease in the number of vessel-associated microglia compared to total IBA-1-positive cells, without a change in the total number of IBA-1-positive cells in the CA1 (Fig. 3 b, c, *p* < 0.05). Consistent with previous reports demonstrating that DE causes no changes in the number of microglia in the substantia nigra [37], *Aif1* (IBA-1) mRNA expression showed no changes in the hippocampus, supporting that the number of microglia is unchanged (Fig. 3 d, *p* > 0.05). These results suggest that DE may impair the microglial association with the neurovasculature, a finding that is different from what occurs with an acute peripheral pro-inflammatory stimulus, such as an IP injection of lipopolysaccharide (LPS), which results in an increase in vessel-associated microglia at 3 h (Supplemental Figure 3, *p* < 0.05). Together, these findings support that DE may disrupt the baseline homeostatic association of microglia with the neurovasculature.

### TREM2 deficiency selectively augments the IL-1β response to DEP

**Fig. 4:**
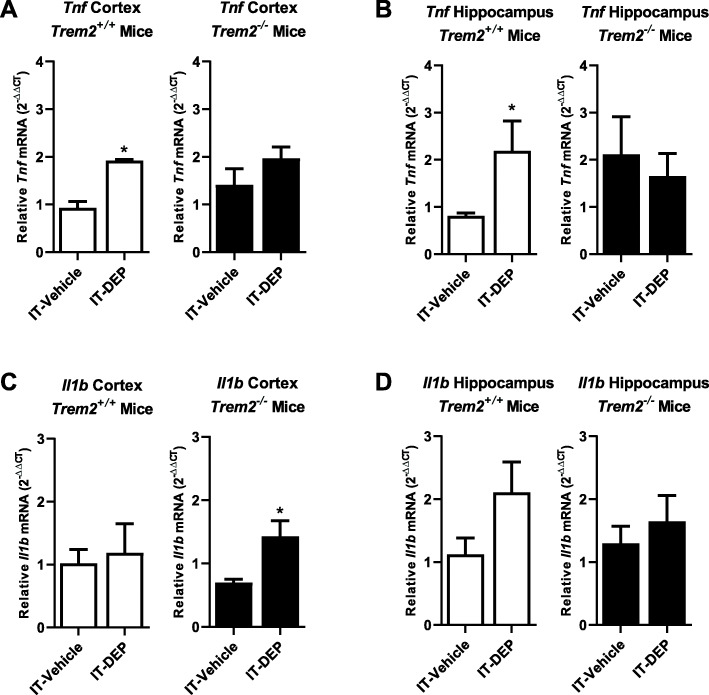
TREM2 deficiency selectively augments cortical *Il1b* gene expression. *Trem2*^*−/−*^ and *Trem2*^*+/+*^ mice were treated with DEP (100 μg/mouse IT, 2× per week, 4 weeks) or vehicle (IT, 2× per week, 4 weeks). mRNA levels were measured by qRT-PCR for *Tnf* (TNFα) (**a**, **b**) and *Il1b* (IL-1β) (**c**, **d**) in the cortex (**a**, **c**) or hippocampus (**b**, **d**) of mice. *p < 0.05, compared with controls (*n* = 3–6)

The impact of TREM2 on neuroinflammation is reported to vary, where TREM2 has been shown to either increase or decrease neuroinflammation depending on the stimulus and context [23, 55]. To discern the role of TREM2 on DE-induced neuroinflammation, we administered DEP by IT instillation for 1 month to *Trem2*^*+/+*^ and *Trem2*^*−/−*^ mice and examined the gene expression of prototypical pro-inflammatory factors. DEP caused a significant increase in *Tnf* (TNFα) expression in both the hippocampus and cortex of *Trem2*^*+/+*^ mice, which was absent in *Trem2*^*−/−*^ mice in the repeated exposure model (Fig. 4 a, b, *p* < 0.05). On the contrary, *Il1b* (IL-1β) increased only in the cortex of *Trem2*^*−/−*^ but not *Trem2*^*+/+*^ mice after administration of DEP (Fig. 4c, *p* < 0.05). *Il1b* expression was not affected in the hippocampus of either genotype (Fig. 4 d, *p* > 0.05) after DEP administration. Interestingly, TREM2^*−/−*^ mice do not have an altered pro-inflammatory response to an IP injection of LPS (Supplemental Figure 4), suggesting that the enhancement of *Il1b* in the cortex of TREM2^*−/−*^ mice may be stimulus dependent. We next examined whether TREM2 was necessary for microglial-vessel association in the cortex and hippocampus of DEP-administered mice. Neither the cortex (Supplemental Figure 5, *p* > 0.05) nor the hippocampus (Supplemental Figure 6, *p* > 0.05) showed changes in the number of vessel-associated microglia, and the expression of *Trem2* by microglia was confirmed by qPCR (Supplemental Figure 7A). These results suggest that loss of TREM2 results in gene and brain region-specific pro-inflammatory response to DEP exposure, but TREM2 does not regulate the microglial association with the neurovasculature.

**Fig. 5:**
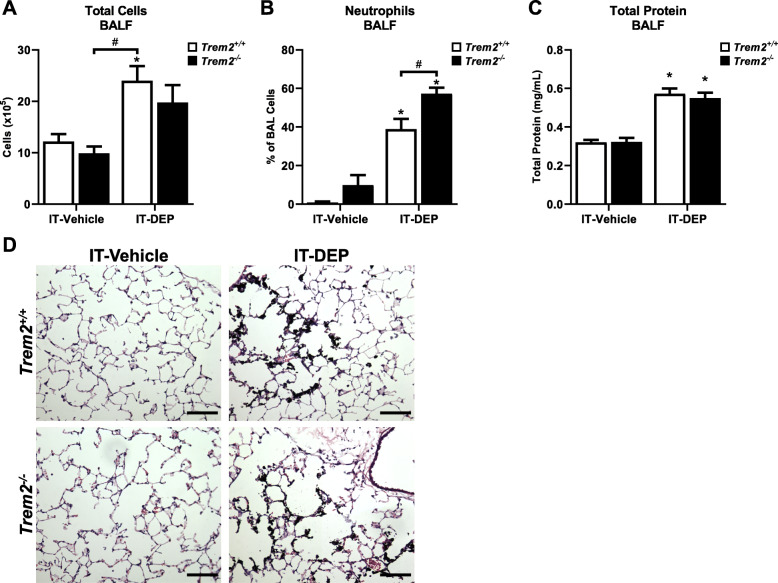
TREM2 deficiency augments neutrophilic pulmonary response to diesel exhaust particles. *Trem2*^*+/+*^ and *Trem2*^*−/−*^ mice were treated with DEP (600 μg/mouse IT, single dose) or vehicle (IT, single dose). Total cell infiltration in bronchoalveolar lavage fluid (BALF) was analyzed by automated cell counter (**a**). Neutrophils were counted from Wright-Giemsa-stained cytospin preparations (**b**). Total protein in BALF was analyzed by BCA assay (**c**). Representative H&E-stained lung sections (**d**). *p < 0.05, compared with controls. ^#^*p* < 0.05 compared between IT-DEP-exposed groups (*n* = 5–6)

In order to further understand the impact of TREM2 deficiency on DEP-induced inflammation, we examined the pulmonary response 24 h after a single intratracheal DEP administration. In concordance with the effects of inhaled DE on pulmonary inflammation [39, 56], DEP-treated mice showed an increase in total cells, neutrophils, and total protein in BALF (Fig. 5 a–c, *p* < 0.05). Further, *Trem2*^*−/−*^ mice showed a greater percentage of neutrophil infiltration into the lung after DEP administration compared to *Trem2*^*+/+*^ mice and that *Trem2*^*−/−*^ mice may have a higher basal level of neutrophils in the alveolar space (Fig. 5 b, *p* < 0.05), which is consistent with prior reports that TREM2^*−/−*^ mice have an augmented pulmonary neutrophil infiltration in response to immunological stimuli [57]. Histopathology of the lungs demonstrates no noticeable difference in alveolar damage between genotypes (Fig. 5d). Expression of *Trem2* by myeloid cells in the BAL was confirmed by qPCR in *Trem2*^*+/+*^ animals (Supplemental Figure 7B). These results suggest that loss of TREM2 may also impact the lung-brain axis by perturbing the DEP-induced pulmonary immune response.

**Fig. 6:**
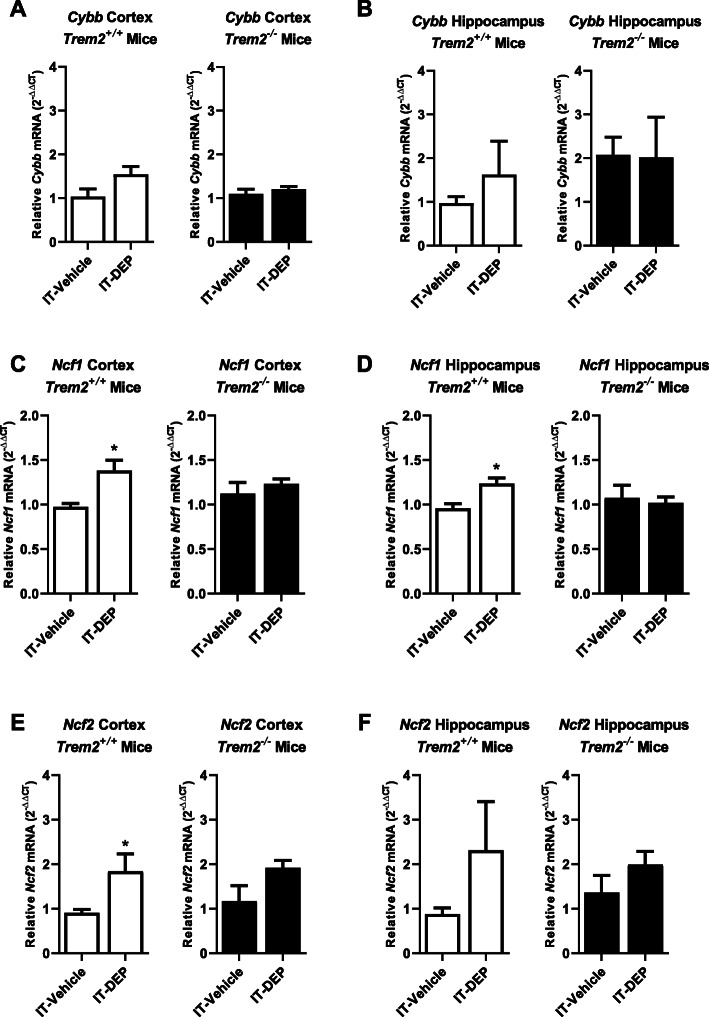
TREM2 regulates diesel exhaust particle-induced NADPH oxidase gene expression. *Trem2*^*−/−*^ and *Trem2*^*+/+*^ mice were treated with DEP (100 μg/mouse IT, 2× per week, 4 weeks) or vehicle (IT, 2× per week, 4 weeks). mRNA levels were measured by qRT-PCR for *Cybb* (gp91^PHOX^) (**a**, **b**), *Ncf1* (p47^PHOX^) (**c**, **d**), and *Ncf2* (p67^PHOX^) in the cortex (**a**, **c**, **e**) or hippocampus (**b**, **d**, **f**). *p < 0.05, compared with controls (*n* = 3–6)

We have previously reported that exposure to high levels of DE increases oxidative stress in the rat brain [37]. To further explore how DE may impact the oxidative state in the CNS, we analyzed the gene expression of several components of the PHOX complex, including *Cybb* (gp91^PHOX^), *Ncf1* (p47^PHOX^), and *Ncf2* (p67^PHOX^). *Cybb* encodes the membrane-bound catalytic subunit of the PHOX complex, while *Ncf1* and *Ncf2* encode two cytosolic components that are required to dock with the membrane-bound components for PHOX activation. The expression of *Cybb* was unchanged in all groups (Fig. 6 a, b, *p* > 0.05). However, *Ncf1* (hippocampus and cortex) and *Ncf2* (cortex) gene expression was increased with DEP treatment in control mice, changes that were absent in *Trem2*^*−/−*^ mice (Fig. 6 c–f, *p* < 0.05). These data suggest that TREM2 is necessary for the DEP-induced upregulation of two key components of the NADPH oxidase complex, p47^PHOX^ and p67^PHOX^, in the mouse brain.

We next explored whether gp91^PHOX^ regulates TREM2 or DE-induced neuroinflammation by utilizing a single administration of DEP to PHOX^+/+^ and PHOX^*−/−*^ animals (Supplemental Figure 8A & B, *p* < 0.05). *Tnf* expression was increased in both genotypes in response to DEP, and *Trem2* was unaffected in either genotype (Supplemental Figure 5 C & D, *p* > 0.05), further supporting that TREM2, but not gp91^PHOX^, is a key regulator of DEP-induced neuroinflammation. Together, these results indicate that loss of TREM2 function selectively upregulates *Il1b* gene expression, but abolishes DEP-induced changes in *Tnf*, *Ncf1*, and *Ncf2* gene expression, demonstrating the important role of TREM2 in DEP-induced neuroinflammation.

## Discussion

Increasing evidence implicates urban air pollution as a contributor to neurodegenerative diseases, such as AD [2, 58], but the underlying mechanisms are poorly understood. While studies have demonstrated that urban air pollution induces neuroinflammation and increases oxidative stress [11, 37, 59], little is known about how air pollution regulates these neuroimmune responses. Here, we demonstrate that DE exposure, a model of urban air pollution, impairs expression of TREM2, dysregulates mRNA expression of markers of the DAM phenotype, and reduces microglial association with the neurovasculature. We also investigated the role of TREM2 in this process, where data indicate that loss of TREM2 augments the pulmonary immune response and modifies the CNS pro-inflammatory response to DEP in a gene- and brain region-specific fashion. These findings support that air pollution perturbs a key disease-modifying pathway in AD, TREM2, to impact the lung-brain axis.

The DE-induced decrease in TREM2 expression in the hippocampus and cortex (Fig. 1) shown in the current study may provide much needed insight into why urban air pollution is associated with AD risk. Inactivating mutations in *TREM2* are known to increase the risk of AD in humans [20, 21, 54], and AD mice deficient in *Trem2* demonstrate an exacerbated AD pathology and increased neuroinflammation at older ages [23]. TREM2 is reported to play key roles during microglial homeostasis [26, 60], as well as during the neurodegenerative process [22]. Further, microglial TREM2 plays important roles in astrocytic synaptic engulfment during development [61], highlighting the broad roles of TREM2 in the CNS. Importantly, TREM2 is necessary for the microglial association with amyloid plaques and the protective barrier response to plaques [23, 27]. Thus, while previous reports have already noted that air pollution can affect plaque load [62], the discovery that DE impairs TREM2 expression suggests that future mechanistic studies should focus on the effect of urban air pollution on amyloid plaque-associated microglia.

Consistent with these findings, our data also support that 1-month DE inhalation dysregulates the expression of markers from both stages of the DAM phenotype (Fig. 2), where dysregulations in *Lpl* (cortex), *Lyz2* (hippocampus), *Cx3cr1* (cortex and hippocampus) were noted. Importantly, TREM2 is a key regulator of the DAM phenotype, which is hypothesized to be a beneficial subset of microglia during the neurodegenerative process [27]. This dysregulation of the DAM phenotype further supports that in response to urban air pollution exposure, microglia may have a more difficult time clearing plaque. Further studies are needed to examine this dysregulation of TREM2 and DAM in an AD model to determine the functional and neuropathological consequences of this dysregulation.

The mechanisms through which urban air pollution can affect microglial responses and neuroinflammation remain a critical question in the field. Recent reports implicate the neurovasculature in the communication between peripheral immune responses and the brain parenchyma [31]. In the current study, we define the impaired microglia-neurovascular interactions in response to DE exposure, where microglia associate less with the neurovasculature after exposure to increasing amounts of DE (Fig. 3). While studies have demonstrated BBB disruption after exposure to pulmonary toxicants in vivo [34], this study is the first to our knowledge to examine the microglial-specific response to the neurovasculature. No changes in the number of microglia were observed in the regions imaged (Fig. 3), suggesting that the change in vascular association was not due to an increase in parenchymal microglia. Further, we observed no changes in *Aif1* mRNA (Fig. 3), supporting the lack of change in the number of microglia in the hippocampus at this time point and these concentrations of DE. Notably, we compared the vascular response of microglia in a DE exposure model to that of a classical peripheral inflammation model, LPS (Supplemental Figure 2), and found the microglia-neurovascular association increased, highlighting that the response to urban air pollution is different than a classic peripheral pro-inflammatory stimulus, like LPS. In fact, these findings support that air pollution exposure may perturb the baseline association of the microglia with the vasculature during homeostasis, where the underlying mechanisms are unknown.

Many forms of urban air pollution trigger neuroinflammation, particularly whole DE and DEP [12, 37, 63], which are hypothesized to be a key mechanism through which air pollution may impact the brain. As such, we also explored the role of TREM2 in the pro-inflammatory gene expression triggered by DEP. Data indicate that *Trem2*^*−/−*^ mice exhibited a unique pattern of neuroinflammation in response to intratracheal administration of DEP, where DEP triggered *Tnf* expression in only control animals, but DEP administration in *Trem2*^*−/−*^ mice resulted in an increase of *Il1b* expression (Fig. 4). Interestingly, these genotype differences were not seen in response to LPS, a classical pro-inflammatory stimulus (Supplemental Figure 4). This observation has important implications for how impaired TREM2 expression or signaling may result in a dysregulated CNS and peripheral immune response to inhaled air pollutants.

Increasing evidence points to the lung-brain axis as an important mechanism of air pollution-induced CNS effects [64, 65]. Importantly, TREM2 plays a key role in myeloid cell function both inside and outside of the CNS [22]. In the current study, these data indicate that *Trem2*^*−/−*^ mice have an increased percentage of neutrophils, which are myeloid cells expressing TREM2, in the lung after DEP administration, when compared to *Trem2*^*+/+*^ mice (Fig. 5). This suggests that TREM2 deficiency may exacerbate the pulmonary neutrophilic response to DEP. Interestingly, this has important implications for the role of TREM2 in the lung-brain axis, where loss of TREM2 may dysregulate pulmonary immune cell infiltration, which could in turn regulate the CNS neuroimmune response. Previous research has demonstrated that DE exposure results in oxidative stress in vivo and in vitro [12, 37]. Importantly, NADPH oxidase (phagocytic oxidase, PHOX), an enzyme complex that is a primary source of microglia-derived ROS, has also been previously implicated as an essential component of the microglial response to DEP [59, 66]. Surprisingly, the current study discovered that mice missing the catalytic subunit of NADPH oxidase, gp91^PHOX^, showed the same CNS cytokine response to DEP as control mice (Supplemental Figure 8), indicating that NADPH oxidase does not regulate cytokine response to DEP in vivo. To further explore how DE/DEP may regulate the oxidative stress in the brain, we also investigated how *Trem2* deficiency affects gene expression of components of the NADPH oxidase system in response to DEP exposure. Data revealed that control mice upregulate two cytosolic components of the NADPH oxidase system (*Ncf1* and *Ncf2*) in response to DEP (Fig. 6), an effect absent in *Trem2*^*−/−*^ mice (Fig. 6), indicating the necessary role of TREM2 in this component of DEP-induced neuroinflammation. Notably, the role of TREM2 in AD is reported to change with age, where TREM2 loss early in disease neuropathology is beneficial in murine AD models, while TREM2 loss is detrimental later in the progression of pathology [23]. Thus, future investigations should focus on the impact of TREM2 on air pollution-induced neuroimmune parameters with increasing age and ongoing AD neuropathology.

## Conclusion

The current study demonstrates that DE exposure impairs TREM2 expression, dysregulates gene expression markers in both stages of the generally beneficial DAM phenotype, and impairs microglia-neurovascular interactions in the brain, pointing to a new mechanism through which urban air pollution may affect CNS diseases, particularly AD. Data suggest that the microglial association with the vasculature may have a TREM2-independent homeostatic function, where perturbation with DE disrupts these baseline cellular interactions, providing new insight into how urban air pollution could affect the brain. This work also revealed a key regulatory role for TREM2 in the pro-inflammatory response to DEP in the lung and the brain, highlighting important neuroimmune consequences for the loss of TREM2 expression. Surprisingly, experiments also showed that deficiency of gp91^PHOX^ (the catalytic subunit of NADPH oxidase) has no effect on DEP-induced neuroinflammation, but instead, TREM2 regulates the DEP-induced gene expression of *Ncf1* and *Ncf2*, genes that encode two cytosolic components of NADPH oxidase. Together, these findings highlight novel mechanisms through which urban air pollution exposure affects key AD-related genes, dysregulates microglial responses, and demonstrates the important role of TREM2 in this process, which has implications for the lung-brain axis and how air pollution exposure may impact and/or augment AD. As such, future studies focusing on the impact of urban air pollution on TREM2-associated Alzheimer’s disease neuropathology will be of pressing interest.

## Supplementary Information

**Additional file 1: Table S1.** Rat Primer Sequences for qPCR. Tumor Necrosis Factor α, TNFα; lipoprotein lipase, LPL; triggering receptor expressed on myeloid cells 2, TREM2; lysozyme, Lyz2; ionized calcium binding adaptor molecule 1, IBA-1; C-X3-C motif chemokine receptor 1, CX3CR1; glyceraldehyde 3-phosphate dehydrogenase, GAPDH; vascular cell adhesion molecule 1, VCAM-1; intracellular adhesion molecule 1, ICAM-1; aquaporin 4, Aqp4; multidrug resistance protein 1b, Mdr1b.

**Additional file 2: Table S2.** Mouse Primer Sequences for qPCR. Tumor Necrosis Factor α, TNFα; interleukin 1 β, IL-1β; neutrophil cytosolic factor 1, Ncf1; neutrophil cytosolic factor 2, Ncf2; cytochrome b-245, beta polypeptide, Cybb; glyceraldehyde 3-phosphate dehydrogenase, GAPDH; triggering receptor expressed on myeloid cells 2, Trem2.

**Additional file 3: Figure S1.** Diesel Exhaust Exposure Fails to Affect Markers of Antioxidant System. WKY rats were exposed to diesel exhaust (0, 50, 150, or 500 μg/m^3^) by inhalation for 4 weeks. Key markers of the endogenous antioxidant system, *Nfel2l* (NRF2) (A) and *Hmox1* (Heme Oxygenase 1) (B) were analyzed by qRT-PCR in the cortex. Data are reported as the mean ± SEM. (n = 7). **Figure S2.** Diesel Exhaust Exposure Fails to Affect Markers of Blood Brain Barrier Function. WKY rats were exposed to diesel exhaust (0, 50, 150, or 500 μg/m^3^) by inhalation for 4 weeks. Key markers of the BBB, *Aqp4* (Aquaporin-4), *Mdr1b* (p-Glycoprotein*)*, *Icam1* (ICAM-1*)*, and *Vcam1* (VCAM-1*)* were analyzed by qRT-PCR in the hippocampus. Data are reported as the mean ± SEM. (n = 7). **Figure S3.** Peripherally Administered LPS Increases Microglial Association with the Vasculature at 3 Hours. C57Bl/6 J mice were administered LPS (5 mg/kg, IP) or saline (IP). The changes in the association of microglia cell bodies with the neurovasculature were assessed with confocal images of microglia (IBA-1, green) and vascular endothelial cells (CD31, red) in the hippocampus (3 slices per brain). Representative maximum intensity projection images are shown (A). Quantification of the number of vessel-associated microglia in CA1 hippocampus (B). *p < 0.05 when compared to control. (n = 3). **Figure S4.** TREM2 Deficiency Does Not Impact Neuroinflammatory Response to Peripherally Administered LPS at 3 Hours. C57Bl/6 J mice were administered LPS (5 mg/kg, IP) or saline (IP). Expression of the pro-inflammatory factors *Tnf* (A), *Il1b* (B), and the cytosolic PHOX component *Ncf1* (C) were analyzed by qRT-PCR in the cortex. Data are reported as the mean ± SEM. *p < 0.05 when compared to *Trem2*^*+/+*^ control. ^#^*p* < 0.05 compared between *Trem2*^*-/-*^ control. (n = 5-6). **Figure S5.** Intratracheal DEP Fails to Affect Microglia-Vessel Association in the Cortex. *Trem2*^*-/-*^ and *Trem2*^*+/+*^ mice were treated with DEP (100 μg/mouse IT, 2x per week, 4 weeks) or vehicle (IT, 2x per week, 4 weeks). The changes in the association of microglia cell bodies with the neurovasculature were assessed with confocal images of microglia (IBA-1, green) and vascular endothelial cells (CD31, red) in the cortex (4 slices per brain). Representative maximum intensity projection images are shown (A). Quantification of the number of vessel-associated microglia in layer V cortex (B). (n = 3-6). **Figure S6.** Intratracheal DEP Fails to Affect Microglia-Vessel Association in the Hippocampus. *Trem2*^*-/-*^ and *Trem2*^*+/+*^ mice were treated with DEP (100 μg/mouse IT, 2x per week, 4 weeks) or vehicle (IT, 2x per week, 4 weeks). The changes in the association of microglia cell bodies with the neurovasculature were assessed with confocal images of microglia (IBA-1, green) and vascular endothelial cells (CD31, red) in the hippocampus (4 slices per brain). Representative maximum intensity projection images are shown (A). Quantification of the number of vessel-associated microglia in CA1 hippocampus (B). (n = 3-6). **Figure S7.** *Trem2* mRNA is Expressed by Isolated Microglia and Immune Cells in Bronchoalveolar Lavage. C57Bl/6 J mice were treated with DEP (600 μg/mouse IT, single dose) or vehicle (50 μL IT). mRNA levels of *Trem2* were measured by qRT-PCR in isolated microglial cells (A) and immune cells collected by bronchoalveolar lavage (B). Data are reported as the mean ± SEM. (n = 3-5). **Figure S8.** gp91^PHOX^ Fails to Regulate DEP-Induced Neuroinflammation. PHOX^-/-^ or PHOX^+/+^ control mice were treated with DEP (600 μg/mouse IT, single dose) or vehicle (50 μL IT). mRNA levels were measured by qRT-PCR for *Tnf* (TNFα) (A,B) and *Trem2* (C,D) in the hippocampus (A,C) and cortex (B,D). Data are reported as the mean ± SEM. *p < 0.05, compared with controls. (n = 3-6).

## Data Availability

The data used and/or analyzed in this study are available from the corresponding author upon reasonable request.

## Abbreviations

DAPI: 2-(4-Amidinophenyl)-6-indolecarbamidine dihydrochloride
AD: Alzheimer’s disease
AQP4: Aquaporin-4
BBB: Blood-brain barrier
BALF: Bronchoalveolar lavage fluid
CNS: Central nervous system
CD31: Cluster of differentiation 31
CX3CR1: C-X3-C motif chemokine receptor 1
CYBB: Cytochrome b-245, beta polypeptide
DE: Diesel exhaust
DEP: Diesel exhaust particles
DAM: Disease-associated microglia
GAPDH: Glyceraldehyde 3-phosphate dehydrogenase
IL-1β: Interleukin 1 beta
ICAM1: Intracellular adhesion molecule 1
IBA-1: Ionized calcium-binding adapter molecule 1
LPS: Lipopolysaccharide
LPL: Lipoprotein lipase
LYZ2: Lysozyme
MDR1B: Multidrug resistance protein 1b
NCF1: Neutrophil cytosolic factor 1
NCF2: Neutrophil cytosolic factor 2
PHOX: Phagocytic oxidase
ROS: Reactive oxygen species
SRM 2975: Standard reference material 2975
TREM2: Triggering receptor expressed on myeloid cells 2
TNFα: Tumor necrosis factor alpha
VCAM1: Vascular cell adhesion molecule 1
