# Supplementary material for: Diesel exhaust impairs TREM2 to dysregulate neuroinflammation
Source: J Neuroinflammation. 2020 Nov 22;17:351. doi: 10.1186/s12974-020-02017-7 (PMC7682066; doi:10.1186/s12974-020-02017-7)
Supplement: Supplementary file 1 — Additional file 1: Table S1. Rat Primer Sequences for qPCR. Tumor Necrosis Factor α, TNFα; lipoprotein lipase, LPL; triggering receptor expressed on myeloid cells 2, TREM2; lysozyme, Lyz2; ionized calcium binding adaptor molecule 1, IBA-1; C-X3-C motif chemokine receptor 1, CX3CR1; glyceraldehyde 3-phosphate dehydrogenase, GAPDH; vascular cell adhesion molecule 1, VCAM-1; intracellular adhesion molecule 1, ICAM-1; aquaporin 4, Aqp4; multidrug resistance protein 1b, Mdr1b. [file 12974_2020_2017_MOESM1_ESM.docx]

**Table S1. Rat Primer Sequences for qPCR.** Tumor Necrosis Factor α, TNFα; lipoprotein lipase, LPL; triggering receptor expressed on myeloid cells 2, TREM2; lysozyme, Lyz2; ionized calcium binding adaptor molecule 1, IBA-1; C-X3-C motif chemokine receptor 1, CX3CR1; glyceraldehyde 3-phosphate dehydrogenase, GAPDH; vascular cell adhesion molecule 1, VCAM-1; intracellular adhesion molecule 1, ICAM-1; aquaporin 4, Aqp4; multidrug resistance protein 1b, Mdr1b.

| \| **Gene** \| **Forward Primer** \| **Reverse Primer** \| \| --- \| --- \| --- \| \| ***Tnf* (TNFα)** \| 5'-ACTGAACTTCGGGGTGATCG-3' \| 5'-CTTGGTGGTTTGCTACGACG-3' \| \| ***Lpl*** \| 5'-TGCCCTAAGGACCCCTGAAG-3' \| 5'-ACATTCCTGTCACCGTCCATC-3' \| \| ***Trem2*** \| 5'-AACTTCAGATCCTACTGGACCC-3' \| 5'-GCAGAACAGAAGTCTTGGTGG-3' \| \| ***Lyz2*** \| 5'-CTGGCAGACTGGGTGTGTTT-3' \| 5'-CCCATAGTCGGTGCTTTGGT-3' \| \| ***Aif1* (IBA-1)** \| 5'-GCCACCAGCGTCTGAGGAGC-3' \| 5'-TCGAGGAAGTGCTTGTTGATCCCA-3' \| \| ***Cx3cr1*** \| 5'-CCCAGCTGCTCAGGACCTCAC-3' \| 5'-TGGTCCCAAAGGCCACGATGT-3' \| \| ***Gapdh* (GAPDH)** \| 5'-CCTGGAGAAACCTGCCAAGTAT-3' \| 5'-AGCCCAGGATGCCCTTTAGT-3' \| \| ***Vcam1* (VCAM-1)** \| 5’-TTTGCAAGAAAAGCCAACATGAAAG-3’ \| 5’-TCTCCAACAGTTCAGACGTTAGC-3’ \| \| ***Icam1* (ICAM-1)** \| 5’-TATGACTCGTGAAAGAAATCAGCTC-3’ \| 5’-CCACAGGTCAGGGTGCTTTCCTCAA-3’ \| \| ***Aqp4* (Aquaporin-4)** \| 5’-TGGGAGTCACCACGGTTCAT-3’ \| 5’-TCACAGCTGGCAAAAATGGTG-3’ \| \| ***Mdr1b* (p-Glycoprotein)** \| 5’-CTGGCAGCTGGGAGACAAAT-3’ \| 5’-GGAGACGTCATCTGTGAGCC-3’ \| | \| **Gene** \| **Forward Primer** \| **Reverse Primer** \| \| --- \| --- \| --- \| \| ***Tnf* (TNFα)** \| 5'-ACTGAACTTCGGGGTGATCG-3' \| 5'-CTTGGTGGTTTGCTACGACG-3' \| \| ***Lpl*** \| 5'-TGCCCTAAGGACCCCTGAAG-3' \| 5'-ACATTCCTGTCACCGTCCATC-3' \| \| ***Trem2*** \| 5'-AACTTCAGATCCTACTGGACCC-3' \| 5'-GCAGAACAGAAGTCTTGGTGG-3' \| \| ***Lyz2*** \| 5'-CTGGCAGACTGGGTGTGTTT-3' \| 5'-CCCATAGTCGGTGCTTTGGT-3' \| \| ***Aif1* (IBA-1)** \| 5'-GCCACCAGCGTCTGAGGAGC-3' \| 5'-TCGAGGAAGTGCTTGTTGATCCCA-3' \| \| ***Cx3cr1*** \| 5'-CCCAGCTGCTCAGGACCTCAC-3' \| 5'-TGGTCCCAAAGGCCACGATGT-3' \| \| ***Gapdh* (GAPDH)** \| 5'-CCTGGAGAAACCTGCCAAGTAT-3' \| 5'-AGCCCAGGATGCCCTTTAGT-3' \| \| ***Vcam1* (VCAM-1)** \| 5’-TTTGCAAGAAAAGCCAACATGAAAG-3’ \| 5’-TCTCCAACAGTTCAGACGTTAGC-3’ \| \| ***Icam1* (ICAM-1)** \| 5’-TATGACTCGTGAAAGAAATCAGCTC-3’ \| 5’-CCACAGGTCAGGGTGCTTTCCTCAA-3’ \| \| ***Aqp4* (Aquaporin-4)** \| 5’-TGGGAGTCACCACGGTTCAT-3’ \| 5’-TCACAGCTGGCAAAAATGGTG-3’ \| \| ***Mdr1b* (p-Glycoprotein)** \| 5’-CTGGCAGCTGGGAGACAAAT-3’ \| 5’-GGAGACGTCATCTGTGAGCC-3’ \| | \| **Gene** \| **Forward Primer** \| **Reverse Primer** \| \| --- \| --- \| --- \| \| ***Tnf* (TNFα)** \| 5'-ACTGAACTTCGGGGTGATCG-3' \| 5'-CTTGGTGGTTTGCTACGACG-3' \| \| ***Lpl*** \| 5'-TGCCCTAAGGACCCCTGAAG-3' \| 5'-ACATTCCTGTCACCGTCCATC-3' \| \| ***Trem2*** \| 5'-AACTTCAGATCCTACTGGACCC-3' \| 5'-GCAGAACAGAAGTCTTGGTGG-3' \| \| ***Lyz2*** \| 5'-CTGGCAGACTGGGTGTGTTT-3' \| 5'-CCCATAGTCGGTGCTTTGGT-3' \| \| ***Aif1* (IBA-1)** \| 5'-GCCACCAGCGTCTGAGGAGC-3' \| 5'-TCGAGGAAGTGCTTGTTGATCCCA-3' \| \| ***Cx3cr1*** \| 5'-CCCAGCTGCTCAGGACCTCAC-3' \| 5'-TGGTCCCAAAGGCCACGATGT-3' \| \| ***Gapdh* (GAPDH)** \| 5'-CCTGGAGAAACCTGCCAAGTAT-3' \| 5'-AGCCCAGGATGCCCTTTAGT-3' \| \| ***Vcam1* (VCAM-1)** \| 5’-TTTGCAAGAAAAGCCAACATGAAAG-3’ \| 5’-TCTCCAACAGTTCAGACGTTAGC-3’ \| \| ***Icam1* (ICAM-1)** \| 5’-TATGACTCGTGAAAGAAATCAGCTC-3’ \| 5’-CCACAGGTCAGGGTGCTTTCCTCAA-3’ \| \| ***Aqp4* (Aquaporin-4)** \| 5’-TGGGAGTCACCACGGTTCAT-3’ \| 5’-TCACAGCTGGCAAAAATGGTG-3’ \| \| ***Mdr1b* (p-Glycoprotein)** \| 5’-CTGGCAGCTGGGAGACAAAT-3’ \| 5’-GGAGACGTCATCTGTGAGCC-3’ \| |
| --- | --- | --- | --- | --- | --- | --- | --- | --- | --- | --- | --- | --- | --- | --- | --- | --- | --- | --- | --- | --- | --- | --- | --- | --- | --- | --- | --- | --- | --- | --- | --- | --- | --- | --- | --- | --- | --- | --- | --- | --- | --- | --- | --- | --- | --- | --- | --- | --- | --- | --- | --- | --- | --- | --- | --- | --- | --- | --- | --- | --- | --- | --- | --- | --- | --- | --- | --- | --- | --- | --- | --- | --- | --- | --- | --- | --- | --- | --- | --- | --- | --- | --- | --- | --- | --- | --- | --- | --- | --- | --- | --- | --- | --- | --- | --- | --- | --- | --- | --- | --- | --- | --- | --- | --- | --- | --- | --- | --- | --- | --- |
| \| **Gene** \| **Forward Primer** \| **Reverse Primer** \| \| --- \| --- \| --- \| \| ***Tnf* (TNFα)** \| 5'-ACTGAACTTCGGGGTGATCG-3' \| 5'-CTTGGTGGTTTGCTACGACG-3' \| \| ***Lpl*** \| 5'-TGCCCTAAGGACCCCTGAAG-3' \| 5'-ACATTCCTGTCACCGTCCATC-3' \| \| ***Trem2*** \| 5'-AACTTCAGATCCTACTGGACCC-3' \| 5'-GCAGAACAGAAGTCTTGGTGG-3' \| \| ***Lyz2*** \| 5'-CTGGCAGACTGGGTGTGTTT-3' \| 5'-CCCATAGTCGGTGCTTTGGT-3' \| \| ***Aif1* (IBA-1)** \| 5'-GCCACCAGCGTCTGAGGAGC-3' \| 5'-TCGAGGAAGTGCTTGTTGATCCCA-3' \| \| ***Cx3cr1*** \| 5'-CCCAGCTGCTCAGGACCTCAC-3' \| 5'-TGGTCCCAAAGGCCACGATGT-3' \| \| ***Gapdh* (GAPDH)** \| 5'-CCTGGAGAAACCTGCCAAGTAT-3' \| 5'-AGCCCAGGATGCCCTTTAGT-3' \| \| ***Vcam1* (VCAM-1)** \| 5’-TTTGCAAGAAAAGCCAACATGAAAG-3’ \| 5’-TCTCCAACAGTTCAGACGTTAGC-3’ \| \| ***Icam1* (ICAM-1)** \| 5’-TATGACTCGTGAAAGAAATCAGCTC-3’ \| 5’-CCACAGGTCAGGGTGCTTTCCTCAA-3’ \| \| ***Aqp4* (Aquaporin-4)** \| 5’-TGGGAGTCACCACGGTTCAT-3’ \| 5’-TCACAGCTGGCAAAAATGGTG-3’ \| \| ***Mdr1b* (p-Glycoprotein)** \| 5’-CTGGCAGCTGGGAGACAAAT-3’ \| 5’-GGAGACGTCATCTGTGAGCC-3’ \| | \| **Gene** \| **Forward Primer** \| **Reverse Primer** \| \| --- \| --- \| --- \| \| ***Tnf* (TNFα)** \| 5'-ACTGAACTTCGGGGTGATCG-3' \| 5'-CTTGGTGGTTTGCTACGACG-3' \| \| ***Lpl*** \| 5'-TGCCCTAAGGACCCCTGAAG-3' \| 5'-ACATTCCTGTCACCGTCCATC-3' \| \| ***Trem2*** \| 5'-AACTTCAGATCCTACTGGACCC-3' \| 5'-GCAGAACAGAAGTCTTGGTGG-3' \| \| ***Lyz2*** \| 5'-CTGGCAGACTGGGTGTGTTT-3' \| 5'-CCCATAGTCGGTGCTTTGGT-3' \| \| ***Aif1* (IBA-1)** \| 5'-GCCACCAGCGTCTGAGGAGC-3' \| 5'-TCGAGGAAGTGCTTGTTGATCCCA-3' \| \| ***Cx3cr1*** \| 5'-CCCAGCTGCTCAGGACCTCAC-3' \| 5'-TGGTCCCAAAGGCCACGATGT-3' \| \| ***Gapdh* (GAPDH)** \| 5'-CCTGGAGAAACCTGCCAAGTAT-3' \| 5'-AGCCCAGGATGCCCTTTAGT-3' \| \| ***Vcam1* (VCAM-1)** \| 5’-TTTGCAAGAAAAGCCAACATGAAAG-3’ \| 5’-TCTCCAACAGTTCAGACGTTAGC-3’ \| \| ***Icam1* (ICAM-1)** \| 5’-TATGACTCGTGAAAGAAATCAGCTC-3’ \| 5’-CCACAGGTCAGGGTGCTTTCCTCAA-3’ \| \| ***Aqp4* (Aquaporin-4)** \| 5’-TGGGAGTCACCACGGTTCAT-3’ \| 5’-TCACAGCTGGCAAAAATGGTG-3’ \| \| ***Mdr1b* (p-Glycoprotein)** \| 5’-CTGGCAGCTGGGAGACAAAT-3’ \| 5’-GGAGACGTCATCTGTGAGCC-3’ \| | \| **Gene** \| **Forward Primer** \| **Reverse Primer** \| \| --- \| --- \| --- \| \| ***Tnf* (TNFα)** \| 5'-ACTGAACTTCGGGGTGATCG-3' \| 5'-CTTGGTGGTTTGCTACGACG-3' \| \| ***Lpl*** \| 5'-TGCCCTAAGGACCCCTGAAG-3' \| 5'-ACATTCCTGTCACCGTCCATC-3' \| \| ***Trem2*** \| 5'-AACTTCAGATCCTACTGGACCC-3' \| 5'-GCAGAACAGAAGTCTTGGTGG-3' \| \| ***Lyz2*** \| 5'-CTGGCAGACTGGGTGTGTTT-3' \| 5'-CCCATAGTCGGTGCTTTGGT-3' \| \| ***Aif1* (IBA-1)** \| 5'-GCCACCAGCGTCTGAGGAGC-3' \| 5'-TCGAGGAAGTGCTTGTTGATCCCA-3' \| \| ***Cx3cr1*** \| 5'-CCCAGCTGCTCAGGACCTCAC-3' \| 5'-TGGTCCCAAAGGCCACGATGT-3' \| \| ***Gapdh* (GAPDH)** \| 5'-CCTGGAGAAACCTGCCAAGTAT-3' \| 5'-AGCCCAGGATGCCCTTTAGT-3' \| \| ***Vcam1* (VCAM-1)** \| 5’-TTTGCAAGAAAAGCCAACATGAAAG-3’ \| 5’-TCTCCAACAGTTCAGACGTTAGC-3’ \| \| ***Icam1* (ICAM-1)** \| 5’-TATGACTCGTGAAAGAAATCAGCTC-3’ \| 5’-CCACAGGTCAGGGTGCTTTCCTCAA-3’ \| \| ***Aqp4* (Aquaporin-4)** \| 5’-TGGGAGTCACCACGGTTCAT-3’ \| 5’-TCACAGCTGGCAAAAATGGTG-3’ \| \| ***Mdr1b* (p-Glycoprotein)** \| 5’-CTGGCAGCTGGGAGACAAAT-3’ \| 5’-GGAGACGTCATCTGTGAGCC-3’ \| |
| \| **Gene** \| **Forward Primer** \| **Reverse Primer** \| \| --- \| --- \| --- \| \| ***Tnf* (TNFα)** \| 5'-ACTGAACTTCGGGGTGATCG-3' \| 5'-CTTGGTGGTTTGCTACGACG-3' \| \| ***Lpl*** \| 5'-TGCCCTAAGGACCCCTGAAG-3' \| 5'-ACATTCCTGTCACCGTCCATC-3' \| \| ***Trem2*** \| 5'-AACTTCAGATCCTACTGGACCC-3' \| 5'-GCAGAACAGAAGTCTTGGTGG-3' \| \| ***Lyz2*** \| 5'-CTGGCAGACTGGGTGTGTTT-3' \| 5'-CCCATAGTCGGTGCTTTGGT-3' \| \| ***Aif1* (IBA-1)** \| 5'-GCCACCAGCGTCTGAGGAGC-3' \| 5'-TCGAGGAAGTGCTTGTTGATCCCA-3' \| \| ***Cx3cr1*** \| 5'-CCCAGCTGCTCAGGACCTCAC-3' \| 5'-TGGTCCCAAAGGCCACGATGT-3' \| \| ***Gapdh* (GAPDH)** \| 5'-CCTGGAGAAACCTGCCAAGTAT-3' \| 5'-AGCCCAGGATGCCCTTTAGT-3' \| \| ***Vcam1* (VCAM-1)** \| 5’-TTTGCAAGAAAAGCCAACATGAAAG-3’ \| 5’-TCTCCAACAGTTCAGACGTTAGC-3’ \| \| ***Icam1* (ICAM-1)** \| 5’-TATGACTCGTGAAAGAAATCAGCTC-3’ \| 5’-CCACAGGTCAGGGTGCTTTCCTCAA-3’ \| \| ***Aqp4* (Aquaporin-4)** \| 5’-TGGGAGTCACCACGGTTCAT-3’ \| 5’-TCACAGCTGGCAAAAATGGTG-3’ \| \| ***Mdr1b* (p-Glycoprotein)** \| 5’-CTGGCAGCTGGGAGACAAAT-3’ \| 5’-GGAGACGTCATCTGTGAGCC-3’ \| | \| **Gene** \| **Forward Primer** \| **Reverse Primer** \| \| --- \| --- \| --- \| \| ***Tnf* (TNFα)** \| 5'-ACTGAACTTCGGGGTGATCG-3' \| 5'-CTTGGTGGTTTGCTACGACG-3' \| \| ***Lpl*** \| 5'-TGCCCTAAGGACCCCTGAAG-3' \| 5'-ACATTCCTGTCACCGTCCATC-3' \| \| ***Trem2*** \| 5'-AACTTCAGATCCTACTGGACCC-3' \| 5'-GCAGAACAGAAGTCTTGGTGG-3' \| \| ***Lyz2*** \| 5'-CTGGCAGACTGGGTGTGTTT-3' \| 5'-CCCATAGTCGGTGCTTTGGT-3' \| \| ***Aif1* (IBA-1)** \| 5'-GCCACCAGCGTCTGAGGAGC-3' \| 5'-TCGAGGAAGTGCTTGTTGATCCCA-3' \| \| ***Cx3cr1*** \| 5'-CCCAGCTGCTCAGGACCTCAC-3' \| 5'-TGGTCCCAAAGGCCACGATGT-3' \| \| ***Gapdh* (GAPDH)** \| 5'-CCTGGAGAAACCTGCCAAGTAT-3' \| 5'-AGCCCAGGATGCCCTTTAGT-3' \| \| ***Vcam1* (VCAM-1)** \| 5’-TTTGCAAGAAAAGCCAACATGAAAG-3’ \| 5’-TCTCCAACAGTTCAGACGTTAGC-3’ \| \| ***Icam1* (ICAM-1)** \| 5’-TATGACTCGTGAAAGAAATCAGCTC-3’ \| 5’-CCACAGGTCAGGGTGCTTTCCTCAA-3’ \| \| ***Aqp4* (Aquaporin-4)** \| 5’-TGGGAGTCACCACGGTTCAT-3’ \| 5’-TCACAGCTGGCAAAAATGGTG-3’ \| \| ***Mdr1b* (p-Glycoprotein)** \| 5’-CTGGCAGCTGGGAGACAAAT-3’ \| 5’-GGAGACGTCATCTGTGAGCC-3’ \| | \| **Gene** \| **Forward Primer** \| **Reverse Primer** \| \| --- \| --- \| --- \| \| ***Tnf* (TNFα)** \| 5'-ACTGAACTTCGGGGTGATCG-3' \| 5'-CTTGGTGGTTTGCTACGACG-3' \| \| ***Lpl*** \| 5'-TGCCCTAAGGACCCCTGAAG-3' \| 5'-ACATTCCTGTCACCGTCCATC-3' \| \| ***Trem2*** \| 5'-AACTTCAGATCCTACTGGACCC-3' \| 5'-GCAGAACAGAAGTCTTGGTGG-3' \| \| ***Lyz2*** \| 5'-CTGGCAGACTGGGTGTGTTT-3' \| 5'-CCCATAGTCGGTGCTTTGGT-3' \| \| ***Aif1* (IBA-1)** \| 5'-GCCACCAGCGTCTGAGGAGC-3' \| 5'-TCGAGGAAGTGCTTGTTGATCCCA-3' \| \| ***Cx3cr1*** \| 5'-CCCAGCTGCTCAGGACCTCAC-3' \| 5'-TGGTCCCAAAGGCCACGATGT-3' \| \| ***Gapdh* (GAPDH)** \| 5'-CCTGGAGAAACCTGCCAAGTAT-3' \| 5'-AGCCCAGGATGCCCTTTAGT-3' \| \| ***Vcam1* (VCAM-1)** \| 5’-TTTGCAAGAAAAGCCAACATGAAAG-3’ \| 5’-TCTCCAACAGTTCAGACGTTAGC-3’ \| \| ***Icam1* (ICAM-1)** \| 5’-TATGACTCGTGAAAGAAATCAGCTC-3’ \| 5’-CCACAGGTCAGGGTGCTTTCCTCAA-3’ \| \| ***Aqp4* (Aquaporin-4)** \| 5’-TGGGAGTCACCACGGTTCAT-3’ \| 5’-TCACAGCTGGCAAAAATGGTG-3’ \| \| ***Mdr1b* (p-Glycoprotein)** \| 5’-CTGGCAGCTGGGAGACAAAT-3’ \| 5’-GGAGACGTCATCTGTGAGCC-3’ \| |
| \| **Gene** \| **Forward Primer** \| **Reverse Primer** \| \| --- \| --- \| --- \| \| ***Tnf* (TNFα)** \| 5'-ACTGAACTTCGGGGTGATCG-3' \| 5'-CTTGGTGGTTTGCTACGACG-3' \| \| ***Lpl*** \| 5'-TGCCCTAAGGACCCCTGAAG-3' \| 5'-ACATTCCTGTCACCGTCCATC-3' \| \| ***Trem2*** \| 5'-AACTTCAGATCCTACTGGACCC-3' \| 5'-GCAGAACAGAAGTCTTGGTGG-3' \| \| ***Lyz2*** \| 5'-CTGGCAGACTGGGTGTGTTT-3' \| 5'-CCCATAGTCGGTGCTTTGGT-3' \| \| ***Aif1* (IBA-1)** \| 5'-GCCACCAGCGTCTGAGGAGC-3' \| 5'-TCGAGGAAGTGCTTGTTGATCCCA-3' \| \| ***Cx3cr1*** \| 5'-CCCAGCTGCTCAGGACCTCAC-3' \| 5'-TGGTCCCAAAGGCCACGATGT-3' \| \| ***Gapdh* (GAPDH)** \| 5'-CCTGGAGAAACCTGCCAAGTAT-3' \| 5'-AGCCCAGGATGCCCTTTAGT-3' \| \| ***Vcam1* (VCAM-1)** \| 5’-TTTGCAAGAAAAGCCAACATGAAAG-3’ \| 5’-TCTCCAACAGTTCAGACGTTAGC-3’ \| \| ***Icam1* (ICAM-1)** \| 5’-TATGACTCGTGAAAGAAATCAGCTC-3’ \| 5’-CCACAGGTCAGGGTGCTTTCCTCAA-3’ \| \| ***Aqp4* (Aquaporin-4)** \| 5’-TGGGAGTCACCACGGTTCAT-3’ \| 5’-TCACAGCTGGCAAAAATGGTG-3’ \| \| ***Mdr1b* (p-Glycoprotein)** \| 5’-CTGGCAGCTGGGAGACAAAT-3’ \| 5’-GGAGACGTCATCTGTGAGCC-3’ \| | \| **Gene** \| **Forward Primer** \| **Reverse Primer** \| \| --- \| --- \| --- \| \| ***Tnf* (TNFα)** \| 5'-ACTGAACTTCGGGGTGATCG-3' \| 5'-CTTGGTGGTTTGCTACGACG-3' \| \| ***Lpl*** \| 5'-TGCCCTAAGGACCCCTGAAG-3' \| 5'-ACATTCCTGTCACCGTCCATC-3' \| \| ***Trem2*** \| 5'-AACTTCAGATCCTACTGGACCC-3' \| 5'-GCAGAACAGAAGTCTTGGTGG-3' \| \| ***Lyz2*** \| 5'-CTGGCAGACTGGGTGTGTTT-3' \| 5'-CCCATAGTCGGTGCTTTGGT-3' \| \| ***Aif1* (IBA-1)** \| 5'-GCCACCAGCGTCTGAGGAGC-3' \| 5'-TCGAGGAAGTGCTTGTTGATCCCA-3' \| \| ***Cx3cr1*** \| 5'-CCCAGCTGCTCAGGACCTCAC-3' \| 5'-TGGTCCCAAAGGCCACGATGT-3' \| \| ***Gapdh* (GAPDH)** \| 5'-CCTGGAGAAACCTGCCAAGTAT-3' \| 5'-AGCCCAGGATGCCCTTTAGT-3' \| \| ***Vcam1* (VCAM-1)** \| 5’-TTTGCAAGAAAAGCCAACATGAAAG-3’ \| 5’-TCTCCAACAGTTCAGACGTTAGC-3’ \| \| ***Icam1* (ICAM-1)** \| 5’-TATGACTCGTGAAAGAAATCAGCTC-3’ \| 5’-CCACAGGTCAGGGTGCTTTCCTCAA-3’ \| \| ***Aqp4* (Aquaporin-4)** \| 5’-TGGGAGTCACCACGGTTCAT-3’ \| 5’-TCACAGCTGGCAAAAATGGTG-3’ \| \| ***Mdr1b* (p-Glycoprotein)** \| 5’-CTGGCAGCTGGGAGACAAAT-3’ \| 5’-GGAGACGTCATCTGTGAGCC-3’ \| | \| **Gene** \| **Forward Primer** \| **Reverse Primer** \| \| --- \| --- \| --- \| \| ***Tnf* (TNFα)** \| 5'-ACTGAACTTCGGGGTGATCG-3' \| 5'-CTTGGTGGTTTGCTACGACG-3' \| \| ***Lpl*** \| 5'-TGCCCTAAGGACCCCTGAAG-3' \| 5'-ACATTCCTGTCACCGTCCATC-3' \| \| ***Trem2*** \| 5'-AACTTCAGATCCTACTGGACCC-3' \| 5'-GCAGAACAGAAGTCTTGGTGG-3' \| \| ***Lyz2*** \| 5'-CTGGCAGACTGGGTGTGTTT-3' \| 5'-CCCATAGTCGGTGCTTTGGT-3' \| \| ***Aif1* (IBA-1)** \| 5'-GCCACCAGCGTCTGAGGAGC-3' \| 5'-TCGAGGAAGTGCTTGTTGATCCCA-3' \| \| ***Cx3cr1*** \| 5'-CCCAGCTGCTCAGGACCTCAC-3' \| 5'-TGGTCCCAAAGGCCACGATGT-3' \| \| ***Gapdh* (GAPDH)** \| 5'-CCTGGAGAAACCTGCCAAGTAT-3' \| 5'-AGCCCAGGATGCCCTTTAGT-3' \| \| ***Vcam1* (VCAM-1)** \| 5’-TTTGCAAGAAAAGCCAACATGAAAG-3’ \| 5’-TCTCCAACAGTTCAGACGTTAGC-3’ \| \| ***Icam1* (ICAM-1)** \| 5’-TATGACTCGTGAAAGAAATCAGCTC-3’ \| 5’-CCACAGGTCAGGGTGCTTTCCTCAA-3’ \| \| ***Aqp4* (Aquaporin-4)** \| 5’-TGGGAGTCACCACGGTTCAT-3’ \| 5’-TCACAGCTGGCAAAAATGGTG-3’ \| \| ***Mdr1b* (p-Glycoprotein)** \| 5’-CTGGCAGCTGGGAGACAAAT-3’ \| 5’-GGAGACGTCATCTGTGAGCC-3’ \| |
| \| **Gene** \| **Forward Primer** \| **Reverse Primer** \| \| --- \| --- \| --- \| \| ***Tnf* (TNFα)** \| 5'-ACTGAACTTCGGGGTGATCG-3' \| 5'-CTTGGTGGTTTGCTACGACG-3' \| \| ***Lpl*** \| 5'-TGCCCTAAGGACCCCTGAAG-3' \| 5'-ACATTCCTGTCACCGTCCATC-3' \| \| ***Trem2*** \| 5'-AACTTCAGATCCTACTGGACCC-3' \| 5'-GCAGAACAGAAGTCTTGGTGG-3' \| \| ***Lyz2*** \| 5'-CTGGCAGACTGGGTGTGTTT-3' \| 5'-CCCATAGTCGGTGCTTTGGT-3' \| \| ***Aif1* (IBA-1)** \| 5'-GCCACCAGCGTCTGAGGAGC-3' \| 5'-TCGAGGAAGTGCTTGTTGATCCCA-3' \| \| ***Cx3cr1*** \| 5'-CCCAGCTGCTCAGGACCTCAC-3' \| 5'-TGGTCCCAAAGGCCACGATGT-3' \| \| ***Gapdh* (GAPDH)** \| 5'-CCTGGAGAAACCTGCCAAGTAT-3' \| 5'-AGCCCAGGATGCCCTTTAGT-3' \| \| ***Vcam1* (VCAM-1)** \| 5’-TTTGCAAGAAAAGCCAACATGAAAG-3’ \| 5’-TCTCCAACAGTTCAGACGTTAGC-3’ \| \| ***Icam1* (ICAM-1)** \| 5’-TATGACTCGTGAAAGAAATCAGCTC-3’ \| 5’-CCACAGGTCAGGGTGCTTTCCTCAA-3’ \| \| ***Aqp4* (Aquaporin-4)** \| 5’-TGGGAGTCACCACGGTTCAT-3’ \| 5’-TCACAGCTGGCAAAAATGGTG-3’ \| \| ***Mdr1b* (p-Glycoprotein)** \| 5’-CTGGCAGCTGGGAGACAAAT-3’ \| 5’-GGAGACGTCATCTGTGAGCC-3’ \| | \| **Gene** \| **Forward Primer** \| **Reverse Primer** \| \| --- \| --- \| --- \| \| ***Tnf* (TNFα)** \| 5'-ACTGAACTTCGGGGTGATCG-3' \| 5'-CTTGGTGGTTTGCTACGACG-3' \| \| ***Lpl*** \| 5'-TGCCCTAAGGACCCCTGAAG-3' \| 5'-ACATTCCTGTCACCGTCCATC-3' \| \| ***Trem2*** \| 5'-AACTTCAGATCCTACTGGACCC-3' \| 5'-GCAGAACAGAAGTCTTGGTGG-3' \| \| ***Lyz2*** \| 5'-CTGGCAGACTGGGTGTGTTT-3' \| 5'-CCCATAGTCGGTGCTTTGGT-3' \| \| ***Aif1* (IBA-1)** \| 5'-GCCACCAGCGTCTGAGGAGC-3' \| 5'-TCGAGGAAGTGCTTGTTGATCCCA-3' \| \| ***Cx3cr1*** \| 5'-CCCAGCTGCTCAGGACCTCAC-3' \| 5'-TGGTCCCAAAGGCCACGATGT-3' \| \| ***Gapdh* (GAPDH)** \| 5'-CCTGGAGAAACCTGCCAAGTAT-3' \| 5'-AGCCCAGGATGCCCTTTAGT-3' \| \| ***Vcam1* (VCAM-1)** \| 5’-TTTGCAAGAAAAGCCAACATGAAAG-3’ \| 5’-TCTCCAACAGTTCAGACGTTAGC-3’ \| \| ***Icam1* (ICAM-1)** \| 5’-TATGACTCGTGAAAGAAATCAGCTC-3’ \| 5’-CCACAGGTCAGGGTGCTTTCCTCAA-3’ \| \| ***Aqp4* (Aquaporin-4)** \| 5’-TGGGAGTCACCACGGTTCAT-3’ \| 5’-TCACAGCTGGCAAAAATGGTG-3’ \| \| ***Mdr1b* (p-Glycoprotein)** \| 5’-CTGGCAGCTGGGAGACAAAT-3’ \| 5’-GGAGACGTCATCTGTGAGCC-3’ \| | \| **Gene** \| **Forward Primer** \| **Reverse Primer** \| \| --- \| --- \| --- \| \| ***Tnf* (TNFα)** \| 5'-ACTGAACTTCGGGGTGATCG-3' \| 5'-CTTGGTGGTTTGCTACGACG-3' \| \| ***Lpl*** \| 5'-TGCCCTAAGGACCCCTGAAG-3' \| 5'-ACATTCCTGTCACCGTCCATC-3' \| \| ***Trem2*** \| 5'-AACTTCAGATCCTACTGGACCC-3' \| 5'-GCAGAACAGAAGTCTTGGTGG-3' \| \| ***Lyz2*** \| 5'-CTGGCAGACTGGGTGTGTTT-3' \| 5'-CCCATAGTCGGTGCTTTGGT-3' \| \| ***Aif1* (IBA-1)** \| 5'-GCCACCAGCGTCTGAGGAGC-3' \| 5'-TCGAGGAAGTGCTTGTTGATCCCA-3' \| \| ***Cx3cr1*** \| 5'-CCCAGCTGCTCAGGACCTCAC-3' \| 5'-TGGTCCCAAAGGCCACGATGT-3' \| \| ***Gapdh* (GAPDH)** \| 5'-CCTGGAGAAACCTGCCAAGTAT-3' \| 5'-AGCCCAGGATGCCCTTTAGT-3' \| \| ***Vcam1* (VCAM-1)** \| 5’-TTTGCAAGAAAAGCCAACATGAAAG-3’ \| 5’-TCTCCAACAGTTCAGACGTTAGC-3’ \| \| ***Icam1* (ICAM-1)** \| 5’-TATGACTCGTGAAAGAAATCAGCTC-3’ \| 5’-CCACAGGTCAGGGTGCTTTCCTCAA-3’ \| \| ***Aqp4* (Aquaporin-4)** \| 5’-TGGGAGTCACCACGGTTCAT-3’ \| 5’-TCACAGCTGGCAAAAATGGTG-3’ \| \| ***Mdr1b* (p-Glycoprotein)** \| 5’-CTGGCAGCTGGGAGACAAAT-3’ \| 5’-GGAGACGTCATCTGTGAGCC-3’ \| |
| \| **Gene** \| **Forward Primer** \| **Reverse Primer** \| \| --- \| --- \| --- \| \| ***Tnf* (TNFα)** \| 5'-ACTGAACTTCGGGGTGATCG-3' \| 5'-CTTGGTGGTTTGCTACGACG-3' \| \| ***Lpl*** \| 5'-TGCCCTAAGGACCCCTGAAG-3' \| 5'-ACATTCCTGTCACCGTCCATC-3' \| \| ***Trem2*** \| 5'-AACTTCAGATCCTACTGGACCC-3' \| 5'-GCAGAACAGAAGTCTTGGTGG-3' \| \| ***Lyz2*** \| 5'-CTGGCAGACTGGGTGTGTTT-3' \| 5'-CCCATAGTCGGTGCTTTGGT-3' \| \| ***Aif1* (IBA-1)** \| 5'-GCCACCAGCGTCTGAGGAGC-3' \| 5'-TCGAGGAAGTGCTTGTTGATCCCA-3' \| \| ***Cx3cr1*** \| 5'-CCCAGCTGCTCAGGACCTCAC-3' \| 5'-TGGTCCCAAAGGCCACGATGT-3' \| \| ***Gapdh* (GAPDH)** \| 5'-CCTGGAGAAACCTGCCAAGTAT-3' \| 5'-AGCCCAGGATGCCCTTTAGT-3' \| \| ***Vcam1* (VCAM-1)** \| 5’-TTTGCAAGAAAAGCCAACATGAAAG-3’ \| 5’-TCTCCAACAGTTCAGACGTTAGC-3’ \| \| ***Icam1* (ICAM-1)** \| 5’-TATGACTCGTGAAAGAAATCAGCTC-3’ \| 5’-CCACAGGTCAGGGTGCTTTCCTCAA-3’ \| \| ***Aqp4* (Aquaporin-4)** \| 5’-TGGGAGTCACCACGGTTCAT-3’ \| 5’-TCACAGCTGGCAAAAATGGTG-3’ \| \| ***Mdr1b* (p-Glycoprotein)** \| 5’-CTGGCAGCTGGGAGACAAAT-3’ \| 5’-GGAGACGTCATCTGTGAGCC-3’ \| | \| **Gene** \| **Forward Primer** \| **Reverse Primer** \| \| --- \| --- \| --- \| \| ***Tnf* (TNFα)** \| 5'-ACTGAACTTCGGGGTGATCG-3' \| 5'-CTTGGTGGTTTGCTACGACG-3' \| \| ***Lpl*** \| 5'-TGCCCTAAGGACCCCTGAAG-3' \| 5'-ACATTCCTGTCACCGTCCATC-3' \| \| ***Trem2*** \| 5'-AACTTCAGATCCTACTGGACCC-3' \| 5'-GCAGAACAGAAGTCTTGGTGG-3' \| \| ***Lyz2*** \| 5'-CTGGCAGACTGGGTGTGTTT-3' \| 5'-CCCATAGTCGGTGCTTTGGT-3' \| \| ***Aif1* (IBA-1)** \| 5'-GCCACCAGCGTCTGAGGAGC-3' \| 5'-TCGAGGAAGTGCTTGTTGATCCCA-3' \| \| ***Cx3cr1*** \| 5'-CCCAGCTGCTCAGGACCTCAC-3' \| 5'-TGGTCCCAAAGGCCACGATGT-3' \| \| ***Gapdh* (GAPDH)** \| 5'-CCTGGAGAAACCTGCCAAGTAT-3' \| 5'-AGCCCAGGATGCCCTTTAGT-3' \| \| ***Vcam1* (VCAM-1)** \| 5’-TTTGCAAGAAAAGCCAACATGAAAG-3’ \| 5’-TCTCCAACAGTTCAGACGTTAGC-3’ \| \| ***Icam1* (ICAM-1)** \| 5’-TATGACTCGTGAAAGAAATCAGCTC-3’ \| 5’-CCACAGGTCAGGGTGCTTTCCTCAA-3’ \| \| ***Aqp4* (Aquaporin-4)** \| 5’-TGGGAGTCACCACGGTTCAT-3’ \| 5’-TCACAGCTGGCAAAAATGGTG-3’ \| \| ***Mdr1b* (p-Glycoprotein)** \| 5’-CTGGCAGCTGGGAGACAAAT-3’ \| 5’-GGAGACGTCATCTGTGAGCC-3’ \| | \| **Gene** \| **Forward Primer** \| **Reverse Primer** \| \| --- \| --- \| --- \| \| ***Tnf* (TNFα)** \| 5'-ACTGAACTTCGGGGTGATCG-3' \| 5'-CTTGGTGGTTTGCTACGACG-3' \| \| ***Lpl*** \| 5'-TGCCCTAAGGACCCCTGAAG-3' \| 5'-ACATTCCTGTCACCGTCCATC-3' \| \| ***Trem2*** \| 5'-AACTTCAGATCCTACTGGACCC-3' \| 5'-GCAGAACAGAAGTCTTGGTGG-3' \| \| ***Lyz2*** \| 5'-CTGGCAGACTGGGTGTGTTT-3' \| 5'-CCCATAGTCGGTGCTTTGGT-3' \| \| ***Aif1* (IBA-1)** \| 5'-GCCACCAGCGTCTGAGGAGC-3' \| 5'-TCGAGGAAGTGCTTGTTGATCCCA-3' \| \| ***Cx3cr1*** \| 5'-CCCAGCTGCTCAGGACCTCAC-3' \| 5'-TGGTCCCAAAGGCCACGATGT-3' \| \| ***Gapdh* (GAPDH)** \| 5'-CCTGGAGAAACCTGCCAAGTAT-3' \| 5'-AGCCCAGGATGCCCTTTAGT-3' \| \| ***Vcam1* (VCAM-1)** \| 5’-TTTGCAAGAAAAGCCAACATGAAAG-3’ \| 5’-TCTCCAACAGTTCAGACGTTAGC-3’ \| \| ***Icam1* (ICAM-1)** \| 5’-TATGACTCGTGAAAGAAATCAGCTC-3’ \| 5’-CCACAGGTCAGGGTGCTTTCCTCAA-3’ \| \| ***Aqp4* (Aquaporin-4)** \| 5’-TGGGAGTCACCACGGTTCAT-3’ \| 5’-TCACAGCTGGCAAAAATGGTG-3’ \| \| ***Mdr1b* (p-Glycoprotein)** \| 5’-CTGGCAGCTGGGAGACAAAT-3’ \| 5’-GGAGACGTCATCTGTGAGCC-3’ \| |
| \| **Gene** \| **Forward Primer** \| **Reverse Primer** \| \| --- \| --- \| --- \| \| ***Tnf* (TNFα)** \| 5'-ACTGAACTTCGGGGTGATCG-3' \| 5'-CTTGGTGGTTTGCTACGACG-3' \| \| ***Lpl*** \| 5'-TGCCCTAAGGACCCCTGAAG-3' \| 5'-ACATTCCTGTCACCGTCCATC-3' \| \| ***Trem2*** \| 5'-AACTTCAGATCCTACTGGACCC-3' \| 5'-GCAGAACAGAAGTCTTGGTGG-3' \| \| ***Lyz2*** \| 5'-CTGGCAGACTGGGTGTGTTT-3' \| 5'-CCCATAGTCGGTGCTTTGGT-3' \| \| ***Aif1* (IBA-1)** \| 5'-GCCACCAGCGTCTGAGGAGC-3' \| 5'-TCGAGGAAGTGCTTGTTGATCCCA-3' \| \| ***Cx3cr1*** \| 5'-CCCAGCTGCTCAGGACCTCAC-3' \| 5'-TGGTCCCAAAGGCCACGATGT-3' \| \| ***Gapdh* (GAPDH)** \| 5'-CCTGGAGAAACCTGCCAAGTAT-3' \| 5'-AGCCCAGGATGCCCTTTAGT-3' \| \| ***Vcam1* (VCAM-1)** \| 5’-TTTGCAAGAAAAGCCAACATGAAAG-3’ \| 5’-TCTCCAACAGTTCAGACGTTAGC-3’ \| \| ***Icam1* (ICAM-1)** \| 5’-TATGACTCGTGAAAGAAATCAGCTC-3’ \| 5’-CCACAGGTCAGGGTGCTTTCCTCAA-3’ \| \| ***Aqp4* (Aquaporin-4)** \| 5’-TGGGAGTCACCACGGTTCAT-3’ \| 5’-TCACAGCTGGCAAAAATGGTG-3’ \| \| ***Mdr1b* (p-Glycoprotein)** \| 5’-CTGGCAGCTGGGAGACAAAT-3’ \| 5’-GGAGACGTCATCTGTGAGCC-3’ \| | \| **Gene** \| **Forward Primer** \| **Reverse Primer** \| \| --- \| --- \| --- \| \| ***Tnf* (TNFα)** \| 5'-ACTGAACTTCGGGGTGATCG-3' \| 5'-CTTGGTGGTTTGCTACGACG-3' \| \| ***Lpl*** \| 5'-TGCCCTAAGGACCCCTGAAG-3' \| 5'-ACATTCCTGTCACCGTCCATC-3' \| \| ***Trem2*** \| 5'-AACTTCAGATCCTACTGGACCC-3' \| 5'-GCAGAACAGAAGTCTTGGTGG-3' \| \| ***Lyz2*** \| 5'-CTGGCAGACTGGGTGTGTTT-3' \| 5'-CCCATAGTCGGTGCTTTGGT-3' \| \| ***Aif1* (IBA-1)** \| 5'-GCCACCAGCGTCTGAGGAGC-3' \| 5'-TCGAGGAAGTGCTTGTTGATCCCA-3' \| \| ***Cx3cr1*** \| 5'-CCCAGCTGCTCAGGACCTCAC-3' \| 5'-TGGTCCCAAAGGCCACGATGT-3' \| \| ***Gapdh* (GAPDH)** \| 5'-CCTGGAGAAACCTGCCAAGTAT-3' \| 5'-AGCCCAGGATGCCCTTTAGT-3' \| \| ***Vcam1* (VCAM-1)** \| 5’-TTTGCAAGAAAAGCCAACATGAAAG-3’ \| 5’-TCTCCAACAGTTCAGACGTTAGC-3’ \| \| ***Icam1* (ICAM-1)** \| 5’-TATGACTCGTGAAAGAAATCAGCTC-3’ \| 5’-CCACAGGTCAGGGTGCTTTCCTCAA-3’ \| \| ***Aqp4* (Aquaporin-4)** \| 5’-TGGGAGTCACCACGGTTCAT-3’ \| 5’-TCACAGCTGGCAAAAATGGTG-3’ \| \| ***Mdr1b* (p-Glycoprotein)** \| 5’-CTGGCAGCTGGGAGACAAAT-3’ \| 5’-GGAGACGTCATCTGTGAGCC-3’ \| | \| **Gene** \| **Forward Primer** \| **Reverse Primer** \| \| --- \| --- \| --- \| \| ***Tnf* (TNFα)** \| 5'-ACTGAACTTCGGGGTGATCG-3' \| 5'-CTTGGTGGTTTGCTACGACG-3' \| \| ***Lpl*** \| 5'-TGCCCTAAGGACCCCTGAAG-3' \| 5'-ACATTCCTGTCACCGTCCATC-3' \| \| ***Trem2*** \| 5'-AACTTCAGATCCTACTGGACCC-3' \| 5'-GCAGAACAGAAGTCTTGGTGG-3' \| \| ***Lyz2*** \| 5'-CTGGCAGACTGGGTGTGTTT-3' \| 5'-CCCATAGTCGGTGCTTTGGT-3' \| \| ***Aif1* (IBA-1)** \| 5'-GCCACCAGCGTCTGAGGAGC-3' \| 5'-TCGAGGAAGTGCTTGTTGATCCCA-3' \| \| ***Cx3cr1*** \| 5'-CCCAGCTGCTCAGGACCTCAC-3' \| 5'-TGGTCCCAAAGGCCACGATGT-3' \| \| ***Gapdh* (GAPDH)** \| 5'-CCTGGAGAAACCTGCCAAGTAT-3' \| 5'-AGCCCAGGATGCCCTTTAGT-3' \| \| ***Vcam1* (VCAM-1)** \| 5’-TTTGCAAGAAAAGCCAACATGAAAG-3’ \| 5’-TCTCCAACAGTTCAGACGTTAGC-3’ \| \| ***Icam1* (ICAM-1)** \| 5’-TATGACTCGTGAAAGAAATCAGCTC-3’ \| 5’-CCACAGGTCAGGGTGCTTTCCTCAA-3’ \| \| ***Aqp4* (Aquaporin-4)** \| 5’-TGGGAGTCACCACGGTTCAT-3’ \| 5’-TCACAGCTGGCAAAAATGGTG-3’ \| \| ***Mdr1b* (p-Glycoprotein)** \| 5’-CTGGCAGCTGGGAGACAAAT-3’ \| 5’-GGAGACGTCATCTGTGAGCC-3’ \| |
| \| **Gene** \| **Forward Primer** \| **Reverse Primer** \| \| --- \| --- \| --- \| \| ***Tnf* (TNFα)** \| 5'-ACTGAACTTCGGGGTGATCG-3' \| 5'-CTTGGTGGTTTGCTACGACG-3' \| \| ***Lpl*** \| 5'-TGCCCTAAGGACCCCTGAAG-3' \| 5'-ACATTCCTGTCACCGTCCATC-3' \| \| ***Trem2*** \| 5'-AACTTCAGATCCTACTGGACCC-3' \| 5'-GCAGAACAGAAGTCTTGGTGG-3' \| \| ***Lyz2*** \| 5'-CTGGCAGACTGGGTGTGTTT-3' \| 5'-CCCATAGTCGGTGCTTTGGT-3' \| \| ***Aif1* (IBA-1)** \| 5'-GCCACCAGCGTCTGAGGAGC-3' \| 5'-TCGAGGAAGTGCTTGTTGATCCCA-3' \| \| ***Cx3cr1*** \| 5'-CCCAGCTGCTCAGGACCTCAC-3' \| 5'-TGGTCCCAAAGGCCACGATGT-3' \| \| ***Gapdh* (GAPDH)** \| 5'-CCTGGAGAAACCTGCCAAGTAT-3' \| 5'-AGCCCAGGATGCCCTTTAGT-3' \| \| ***Vcam1* (VCAM-1)** \| 5’-TTTGCAAGAAAAGCCAACATGAAAG-3’ \| 5’-TCTCCAACAGTTCAGACGTTAGC-3’ \| \| ***Icam1* (ICAM-1)** \| 5’-TATGACTCGTGAAAGAAATCAGCTC-3’ \| 5’-CCACAGGTCAGGGTGCTTTCCTCAA-3’ \| \| ***Aqp4* (Aquaporin-4)** \| 5’-TGGGAGTCACCACGGTTCAT-3’ \| 5’-TCACAGCTGGCAAAAATGGTG-3’ \| \| ***Mdr1b* (p-Glycoprotein)** \| 5’-CTGGCAGCTGGGAGACAAAT-3’ \| 5’-GGAGACGTCATCTGTGAGCC-3’ \| | \| **Gene** \| **Forward Primer** \| **Reverse Primer** \| \| --- \| --- \| --- \| \| ***Tnf* (TNFα)** \| 5'-ACTGAACTTCGGGGTGATCG-3' \| 5'-CTTGGTGGTTTGCTACGACG-3' \| \| ***Lpl*** \| 5'-TGCCCTAAGGACCCCTGAAG-3' \| 5'-ACATTCCTGTCACCGTCCATC-3' \| \| ***Trem2*** \| 5'-AACTTCAGATCCTACTGGACCC-3' \| 5'-GCAGAACAGAAGTCTTGGTGG-3' \| \| ***Lyz2*** \| 5'-CTGGCAGACTGGGTGTGTTT-3' \| 5'-CCCATAGTCGGTGCTTTGGT-3' \| \| ***Aif1* (IBA-1)** \| 5'-GCCACCAGCGTCTGAGGAGC-3' \| 5'-TCGAGGAAGTGCTTGTTGATCCCA-3' \| \| ***Cx3cr1*** \| 5'-CCCAGCTGCTCAGGACCTCAC-3' \| 5'-TGGTCCCAAAGGCCACGATGT-3' \| \| ***Gapdh* (GAPDH)** \| 5'-CCTGGAGAAACCTGCCAAGTAT-3' \| 5'-AGCCCAGGATGCCCTTTAGT-3' \| \| ***Vcam1* (VCAM-1)** \| 5’-TTTGCAAGAAAAGCCAACATGAAAG-3’ \| 5’-TCTCCAACAGTTCAGACGTTAGC-3’ \| \| ***Icam1* (ICAM-1)** \| 5’-TATGACTCGTGAAAGAAATCAGCTC-3’ \| 5’-CCACAGGTCAGGGTGCTTTCCTCAA-3’ \| \| ***Aqp4* (Aquaporin-4)** \| 5’-TGGGAGTCACCACGGTTCAT-3’ \| 5’-TCACAGCTGGCAAAAATGGTG-3’ \| \| ***Mdr1b* (p-Glycoprotein)** \| 5’-CTGGCAGCTGGGAGACAAAT-3’ \| 5’-GGAGACGTCATCTGTGAGCC-3’ \| | \| **Gene** \| **Forward Primer** \| **Reverse Primer** \| \| --- \| --- \| --- \| \| ***Tnf* (TNFα)** \| 5'-ACTGAACTTCGGGGTGATCG-3' \| 5'-CTTGGTGGTTTGCTACGACG-3' \| \| ***Lpl*** \| 5'-TGCCCTAAGGACCCCTGAAG-3' \| 5'-ACATTCCTGTCACCGTCCATC-3' \| \| ***Trem2*** \| 5'-AACTTCAGATCCTACTGGACCC-3' \| 5'-GCAGAACAGAAGTCTTGGTGG-3' \| \| ***Lyz2*** \| 5'-CTGGCAGACTGGGTGTGTTT-3' \| 5'-CCCATAGTCGGTGCTTTGGT-3' \| \| ***Aif1* (IBA-1)** \| 5'-GCCACCAGCGTCTGAGGAGC-3' \| 5'-TCGAGGAAGTGCTTGTTGATCCCA-3' \| \| ***Cx3cr1*** \| 5'-CCCAGCTGCTCAGGACCTCAC-3' \| 5'-TGGTCCCAAAGGCCACGATGT-3' \| \| ***Gapdh* (GAPDH)** \| 5'-CCTGGAGAAACCTGCCAAGTAT-3' \| 5'-AGCCCAGGATGCCCTTTAGT-3' \| \| ***Vcam1* (VCAM-1)** \| 5’-TTTGCAAGAAAAGCCAACATGAAAG-3’ \| 5’-TCTCCAACAGTTCAGACGTTAGC-3’ \| \| ***Icam1* (ICAM-1)** \| 5’-TATGACTCGTGAAAGAAATCAGCTC-3’ \| 5’-CCACAGGTCAGGGTGCTTTCCTCAA-3’ \| \| ***Aqp4* (Aquaporin-4)** \| 5’-TGGGAGTCACCACGGTTCAT-3’ \| 5’-TCACAGCTGGCAAAAATGGTG-3’ \| \| ***Mdr1b* (p-Glycoprotein)** \| 5’-CTGGCAGCTGGGAGACAAAT-3’ \| 5’-GGAGACGTCATCTGTGAGCC-3’ \| |
