# Supplementary figures and images for: Diesel exhaust impairs TREM2 to dysregulate neuroinflammation
Source: J Neuroinflammation. 2020 Nov 22;17:351. doi: 10.1186/s12974-020-02017-7 (PMC7682066; doi:10.1186/s12974-020-02017-7)

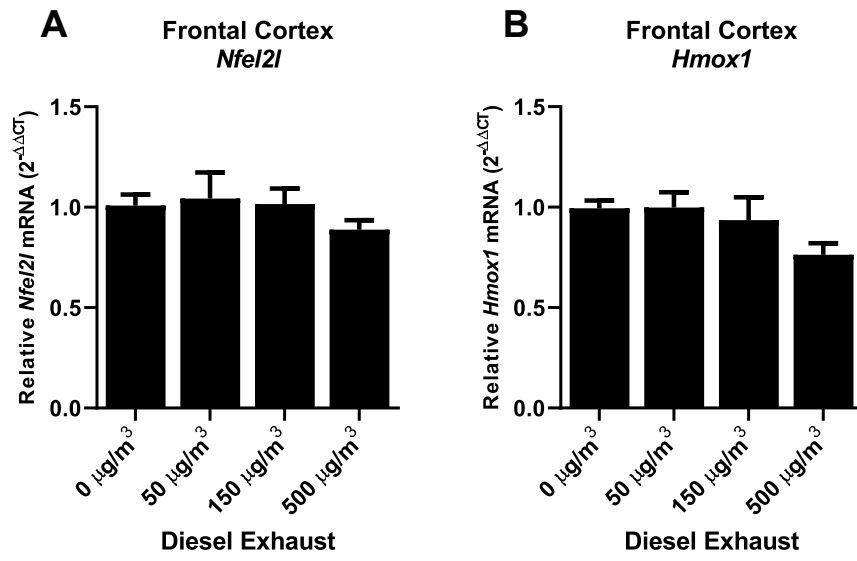

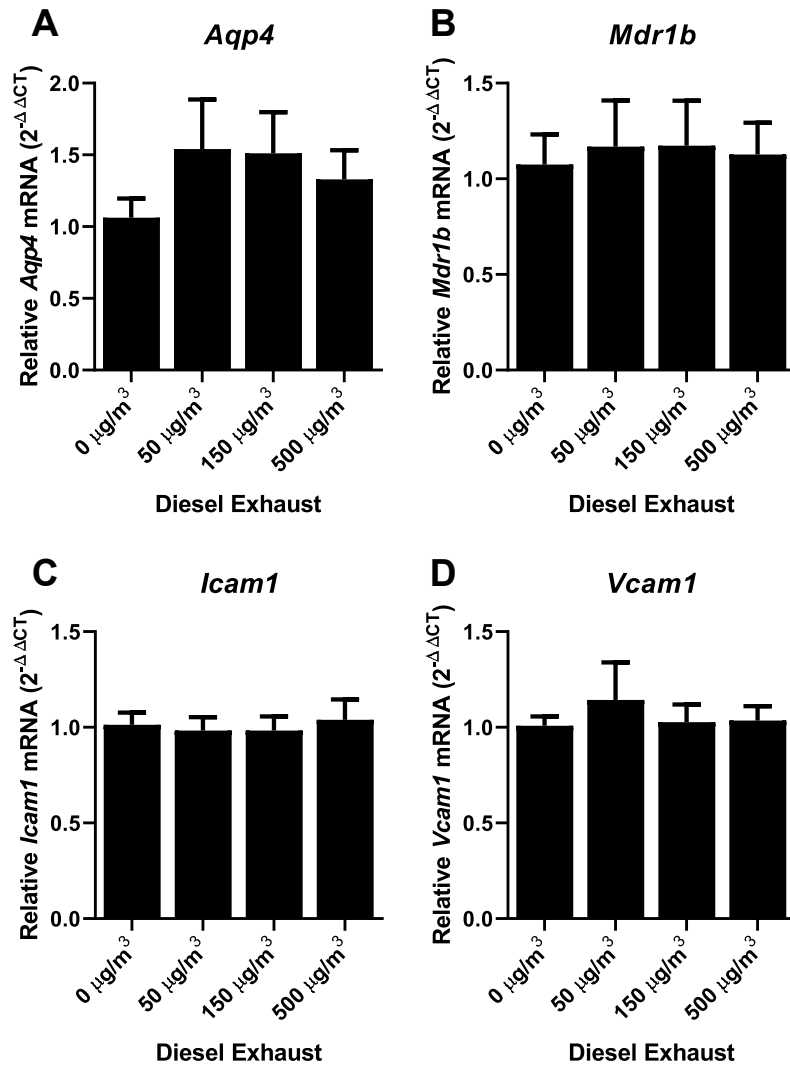

**A**

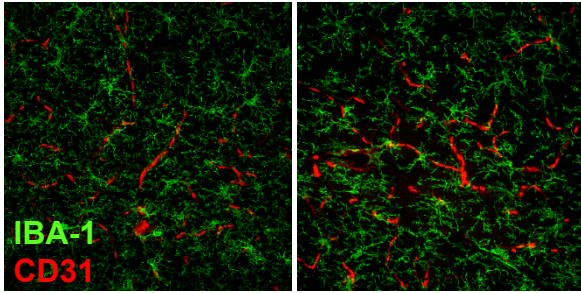

**B**

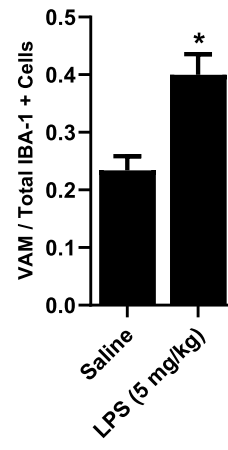

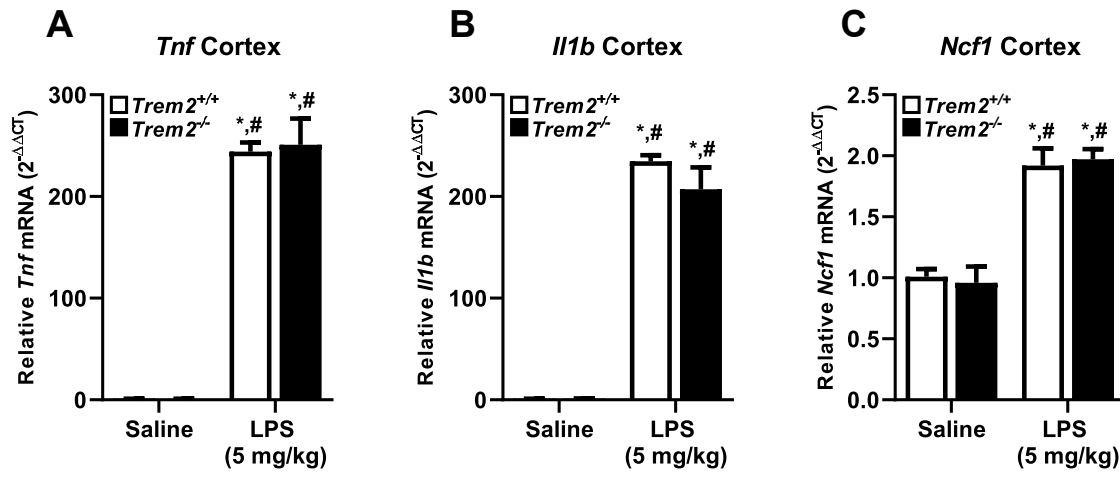

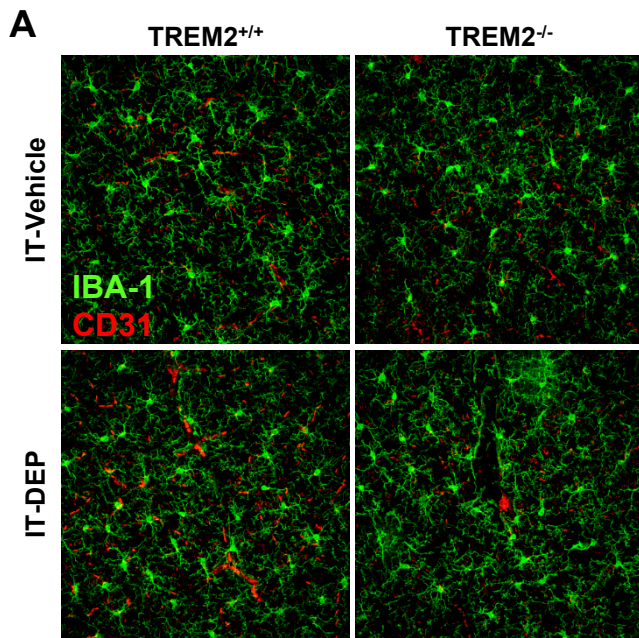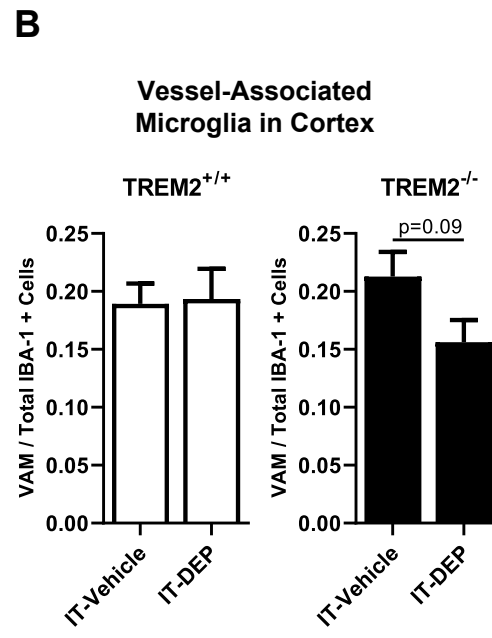

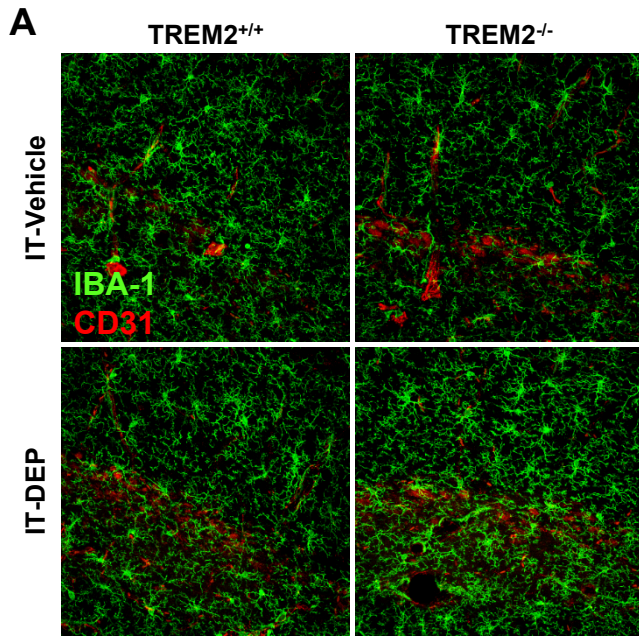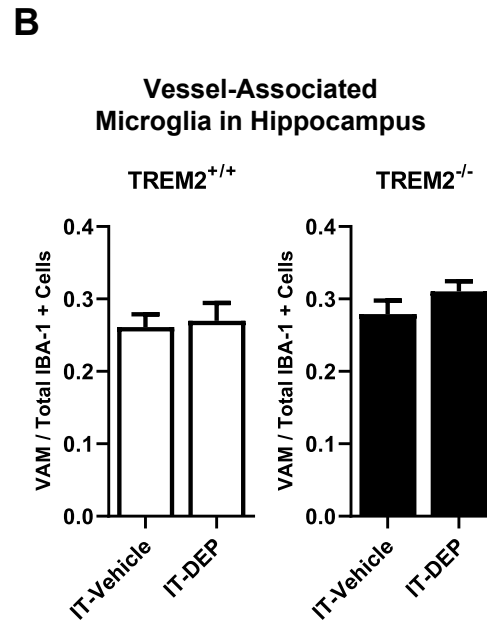

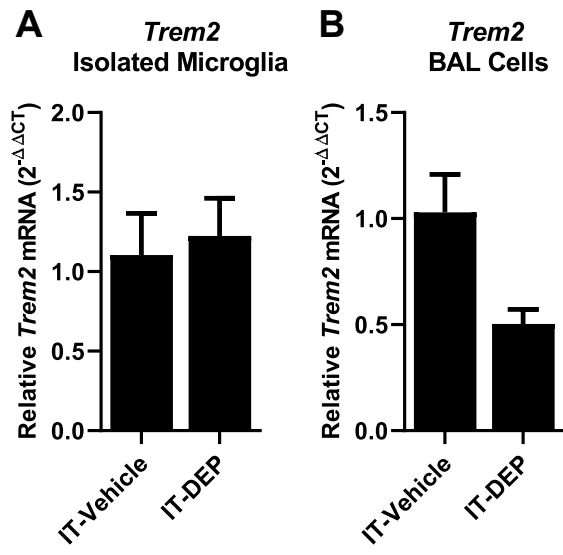

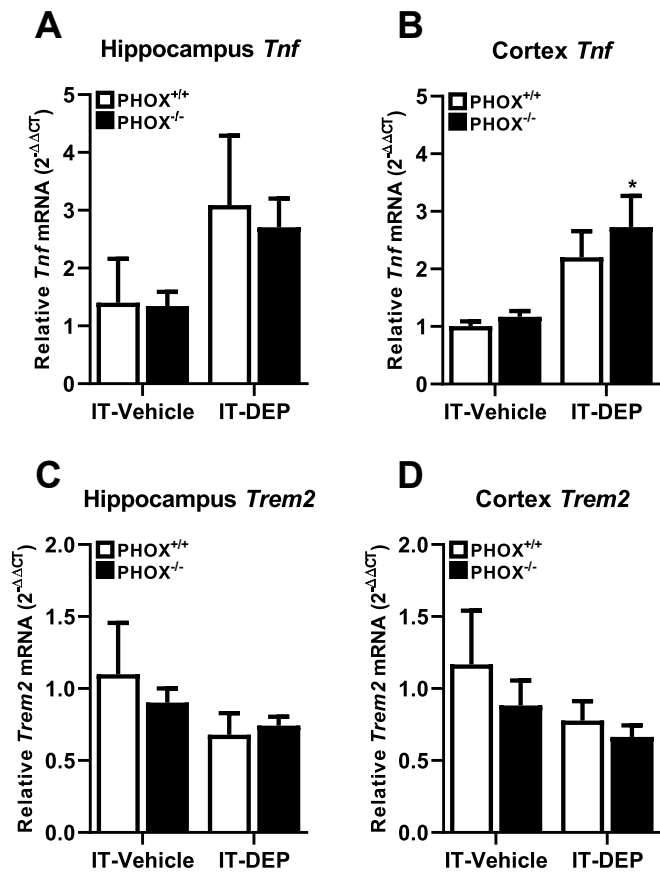

Supplement: Supplementary file 3 — Additional file 3: Figure S1. Diesel Exhaust Exposure Fails to Affect Markers of Antioxidant System. WKY rats were exposed to diesel exhaust (0, 50, 150, or 500 μg/m3) by inhalation for 4 weeks. Key markers of the endogenous antioxidant system, Nfel2l (NRF2) (A) and Hmox1 (Heme Oxygenase 1) (B) were analyzed by qRT-PCR in the cortex. Data are reported as the mean ± SEM. (n = 7). Figure S2. Diesel Exhaust Exposure Fails to Affect Markers of Blood Brain Barrier Function. WKY rats were exposed to diesel exhaust (0, 50, 150, or 500 μg/m3) by inhalation for 4 weeks. Key markers of the BBB, Aqp4 (Aquaporin-4), Mdr1b (p-Glycoprotein), Icam1 (ICAM-1), and Vcam1 (VCAM-1) were analyzed by qRT-PCR in the hippocampus. Data are reported as the mean ± SEM. (n = 7). Figure S3. Peripherally Administered LPS Increases Microglial Association with the Vasculature at 3 Hours. C57Bl/6 J mice were administered LPS (5 mg/kg, IP) or saline (IP). The changes in the association of microglia cell bodies with the neurovasculature were assessed with confocal images of microglia (IBA-1, green) and vascular endothelial cells (CD31, red) in the hippocampus (3 slices per brain). Representative maximum intensity projection images are shown (A). Quantification of the number of vessel-associated microglia in CA1 hippocampus (B). *p < 0.05 when compared to control. (n = 3). Figure S4. TREM2 Deficiency Does Not Impact Neuroinflammatory Response to Peripherally Administered LPS at 3 Hours. C57Bl/6 J mice were administered LPS (5 mg/kg, IP) or saline (IP). Expression of the pro-inflammatory factors Tnf (A), Il1b (B), and the cytosolic PHOX component Ncf1 (C) were analyzed by qRT-PCR in the cortex. Data are reported as the mean ± SEM. *p < 0.05 when compared to Trem2+/+ control. #p < 0.05 compared between Trem2-/- control. (n = 5-6). Figure S5. Intratracheal DEP Fails to Affect Microglia-Vessel Association in the Cortex. Trem2-/- and Trem2+/+ mice were treated with DEP (100 μg/mouse IT, 2x per week, 4 week [file 12974_2020_2017_MOESM3_ESM.pdf]
